# Plasma Functionalization of Silica Bilayer Polymorphs

**DOI:** 10.1021/acsami.2c11491

**Published:** 2022-10-18

**Authors:** Mauricio J. Prieto, Thomas Mullan, Weiming Wan, Liviu C. Tănase, Lucas de Souza Caldas, Shamil Shaikhutdinov, Joachim Sauer, Denis Usvyat, Thomas Schmidt, Beatriz Roldan Cuenya

**Affiliations:** † Department of Interface Science, Fritz-Haber-Institut der Max-Planck-Gesellschaft, Faradayweg 4-6, 14195Berlin, Germany; ‡ Institut für Chemie, Humboldt-Universität zu Berlin, Unter den Linden 6, 10099Berlin, Germany

**Keywords:** ultrathin silica film, crystalline, vitreous, plasma functionalization, hydroxyl, hydride

## Abstract

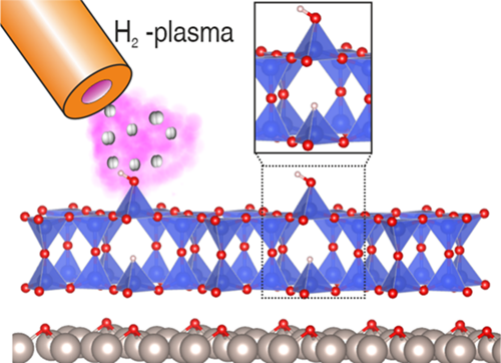

Ultrathin silica films are considered suitable two-dimensional model systems for the study of fundamental chemical and physical properties of all-silica zeolites and their derivatives, as well as novel supports for the stabilization of single atoms. In the present work, we report the creation of a new model catalytic support based on the surface functionalization of different silica bilayer (BL) polymorphs with well-defined atomic structures. The functionalization is carried out by means of in situ H-plasma treatments at room temperature. Low energy electron diffraction and microscopy data indicate that the atomic structure of the films remains unchanged upon treatment. Comparing the experimental results (photoemission and infrared absorption spectra) with density functional theory simulations shows that H_2_ is added via the heterolytic dissociation of an interlayer Si–O–Si siloxane bond and the subsequent formation of a hydroxyl and a hydride group in the top and bottom layers of the silica film, respectively. Functionalization of the silica films constitutes the first step into the development of a new type of model system of single-atom catalysts where metal atoms with different affinities for the functional groups can be anchored in the SiO_2_ matrix in well-established positions. In this way, synergistic and confinement effects between the active centers can be studied in a controlled manner.

## Introduction

SiO_2_-based materials are widely used in many fields of application, such as in electronics,^1^ in the food industry,^2^ as an additive in polymer composite materials,^3^ in heterogeneous^4^ and electro-catalysis,^5^ and in photochemistry.^6^ In many cases, the surface properties of these widely different materials are at the center of their application. For instance, the properties of the Si–SiO_2_ interface in silicon junctions have a major impact on the working efficiency of solar cells.^7^ For this reason, the functionalization of silicon surfaces has been extensively investigated in the past decades to avoid side processes that might be detrimental to the device efficiency and widen the applicability of these surfaces.^8^

Particularly in the field of catalysis, the surface chemical and electronic properties of the various silica-based materials constitute the core of renewed industrial and academic interest.^9,10^ The surface termination of different forms of silica has a crucial effect on their adsorptive properties, e.g., for binding biomolecules.^11^ Moreover, two-dimensional (2D) all-silica zeolites^12^ are used for gas separation in membranes,^13^ and the surface properties of silica nanoclusters play a role in the formation of interstellar silicate dust.^14^ Moreover, the surface termination can impact catalyst performance in various reaction environments. For instance, the strong covalent interactions between the active phase and the support make it possible to tune the reaction selectivity by stabilization of certain specific phases in complex systems. One example is the covalent stabilization of chromium in the Cr/SiO_2_ planar model system of the industrially relevant Phillips catalyst used in the large-scale production of polyethylene.^15^ Another example is the introduction of silica in Co-based catalysts to render an increased selectivity toward methanol synthesis in the hydrogenation of CO_2_. In the latter case, the formation of Co–O–SiO_*n*_ linkages has been proposed to be responsible for the change in selectivity due to the stabilization of the *CH_3_O intermediate/precursor.^16^ Moreover, functionalization of the support can greatly improve the long-term stability of the active phase of the catalyst by preventing processes such as sintering.^17^

In the past decade, a new approach to finding more active materials was proposed, with the main advantage of drastically decreasing the loading of the active components that are often in the form of expensive metals of limited availability. The synthesis and future successful use of this new class of materials, known as single-atom catalysts (SACs), have as pre-requisite being able to design suitable supports that allow proper anchoring/stabilization of the highly dispersed atoms. In the past, this has been attempted on various substrates, including reducible oxides,^18^ 2D dichalcogenides,^19^ defective metal substrates,^20^ and organic frameworks.^21^ Nonetheless, due to the decreased conductivity of some of these systems, most of them are unsuitable for electrochemical applications, where the field of SACs is currently emerging. The present study has overcome this problem by creating an ultrathin SiO_2_ film deposited on a conductive substrate.

Moreover, two key parameters are important variables in SAC-based materials: (i) the local coordination environment of the single-atom site (steric and coordination geometry requirements) and (ii) the local charge density on the single atom as a function of the coordinating environment from the support. These parameters have proven to be very important in determining not only the activity but also the selectivity of SACs.^22^ For instance, it has been shown by Shi et al.^19^ that the local environment in Pt SACs can modulate their electrocatalytic response toward the hydrogen evolution reaction. More specifically, changes in the oxidation state of the active Pt centers were found to strongly affect the adsorption/desorption energy of OH and H species and the overall water splitting process. However, a fundamental understanding of the acting mechanism behind the distinctive reactivity of SACs is still lacking. In this respect, it becomes relevant to develop a new type of support that can offer a well-defined structure and tunable anchoring sites that allow the investigation of local geometric/steric and electronic effects in this new kind of catalyst.

In the former context, the SiO_2_ bilayer (BL) system stands out as an excellent candidate, provided that anchoring points for active centers can be carefully manipulated within the structure. Model SiO_2_ BL films have been reported supported on transition metal (TM) substrates. Different BL films^23−25^ can be prepared, with well-defined 2D structures. Among all known polymorphs, the crystalline and vitreous SiO_2_ BLs have received most of the attention because they are chemically detached from the Ru support and only weakly interact with it via van der Waals forces. Second, both can exist (and coexist) on Ru(0001), provided that the right experimental conditions are met.^26^

In terms of chemical stability, the BL system has proven to be rather robust, possibly because of the saturated and self-contained nature of the Si–O bonds. The vitreous and crystalline BLs consist of SiO_4_ tetrahedral building units connected by Si–O–Si bonds forming contiguous sheets interconnected by O bridging bonds. The registry between the two layers is maintained, while all bonds are saturated. Changing this robust structure is a complicated task, and, in the specific case of hydroxylation, the key aspect seems to be opening the very stable saturated siloxane bonds to create active sites where functional groups can be anchored.

Two different recipes for the functionalization of silica BLs have been reported rather recently. On the one hand, the direct exposure of SiO_2_ BL/Ru(0001) samples to solutions with different pH was studied.^27^ The authors found that, besides the functionalization of the film, its dissolution occurs rather quickly once the hydroxyl anions in the solution attack the film. These results appear to align with results found in aqueous solutions of colloidal silica nanoparticles.^28^

On the other hand, it has been reported that hydroxylation of the silica BL is possible by adsorbing H_2_O at 100 K and subsequent desorption.^29^ Electron bombardment at low temperatures of an ice-covered SiO_2_ BL can increase the concentration of OH groups after the heating step, reinforcing the idea of the siloxane bond activation requirement.^30^ Isotopic labeling experiments proved an oxygen exchange between water molecules at the interface between ice and silica, thus suggesting a certain dynamicity of the siloxane bond breaking/formation. It is important to mention that the crystalline structure exhibited is preserved after the treatments discussed above, except, of course, for the partial film dissolution reported. However, estimations of the amount of −OH groups produced by the latter recipe indicate that their surface concentration can be as low as 0.4 nm^–2^ (i.e., an OH maximum concentration of 2.5%) based on infrared absorption spectra (IRAS) and scanning tunneling microscopy (STM) measurements.^15^ It is important to highlight that both methods described result only in the formation of hydroxyl groups, with possible variation in the position of the OH groups depending on the local structure of the SiO_2_ film.

In the present paper, we describe an entirely new path for the functionalization of the SiO_2_ BL system by means of ultrahigh vacuum (UHV) hydrogen plasma treatments. The use of plasma in catalysis is a relatively unexplored topic, with the focus being mainly on the activation of the catalysts (see ref (31) and references therein) or the reactant molecules that may not be accessible otherwise through thermal routes.^32^ In this sense, the main effect of plasma-assisted catalysis is the possibility of activating strong and stable bonds present either in the catalyst phase or the reactant molecules in less demanding experimental conditions. It is this aspect that makes the plasma application particularly interesting for the functionalization of silica BL films.

A simple consideration of the working principle of a plasma source renders a somewhat limited number of reactive species when operated at relatively low temperatures (300–1300 K). In the case of operation in a H_2_ atmosphere, mostly H_2_^+^ is expected to be the reactive species at temperatures relevant to our experiments (∼300 K).^33^ Therefore, its addition to the silica BL structure is anticipated to follow a heterolytic dissociation of the Si–O–Si bond. Thus, bifunctionalization is expected with neighboring −H and −OH groups formed in the process. Here, a combination of experimental techniques and density functional theory (DFT) modeling allowed us to identify the specific sites in the structures more susceptible to functionalization and the final distribution of the functional groups. The creation of two neighboring functional groups upon plasma treatment at well-defined positions in the BL allows the possibility of its application as a model system for the anchoring of different TMs in the study of the fundamental properties of SACs. Thus, important aspects such as long-term stability under harsh (thermal^34^ and electrochemical^35^) reaction conditions and local electronic and geometric effects can be tackled in a controlled environment. Our bifunctionalization approach represents the first steps into the development of a new type of model system offering coexisting chemically different active sites with a well-defined structure and position, aiming at the fundamental understanding of the properties mentioned above, as well as possible confinement^36^ and synergistic^37^ effects.

## Results and Discussion

**Figure 1:**
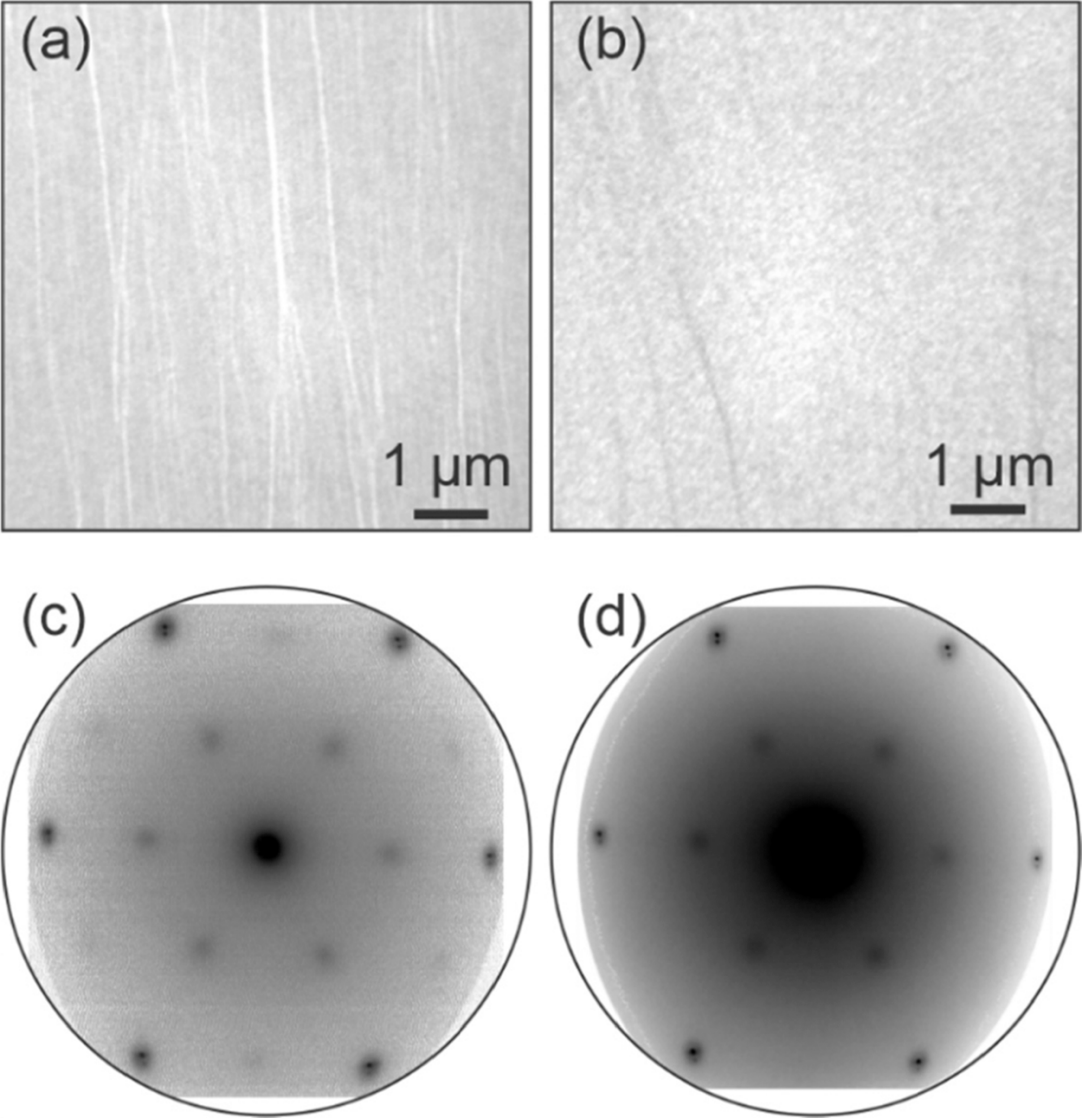
(a,b) LEEM images and (c,d) LEED patterns of the SiO_2_ BL/Ru(0001) samples in the (a,c) pristine state and after (b,d) 64 min of H-plasma exposure. Electron energy: 42 eV.

To determine the effect of the H_2_ plasma treatment, an accumulative exposure approach was applied, with times varying between 1 and 64 min while keeping all other operation parameters of the plasma source constant (e.g., the anode current and voltage—see the Experimental Section for more details). Figure 1 shows a collection of low energy electron microscopy (LEEM) images and diffraction (LEED) patterns recorded for the pristine crystalline state (a,c) and after being exposed to 64 min of H_2_ plasma treatment (b,d). The LEEM images indicate that the film does not undergo extensive reconstruction/degradation after the plasma treatment, as judged from the lack of three-dimensional structure formation at the mesoscopic scale. Only changes in the contrast at step bunches are observed (parallel lines running across the images), a phenomenon already known from the removal of interfacial oxygen (O_Ru_).^38^

The diffraction patterns collected for the pristine and 64 min H-plasma-treated crystalline SiO_2_ BL prove that the atomic order of the film is preserved, as indicated by the prevalence of the (2 × 2) spots. The slight decrease in the intensity of these spots is caused by the removal of interfacial O through the formation of H_2_O molecules once it reacts with the reactive hydrogen species present in the plasma, as it has been reported previously for thermal routes.^25^ As demonstrated in our previous work, a 3O layer is formed on the ruthenium support under the experimental conditions used for the preparation of the pristine film, generating thus virtually the same LEED pattern as for the crystalline silica film.

Unequivocal assignment of these spots is made on the basis that this reconstruction only develops in the LEED pattern while cooling down the sample below 560 K, the temperature at which a disordered to order transition of the interfacial O_Ru_ layer has been observed. For more details, the readers are referred to a previous publication in the subject.^25^ It is important to mention that all exposures to the H-plasma yield the same result in terms of structure preservation. The most striking observation is the increase of the background signal in the LEED pattern, in detriment of the intensity of the diffraction features belonging to the crystalline and vitreous silica films ((2 × 2) or ring, respectively). This suggests that irradiation of the silica BL polymorphs with the H-plasma results in the creation of local defects in the BL, thus maintaining the overall 2D arrangement. The complete series of LEEM images and LEED patterns after successive plasma exposure can be found in the Supporting Information (see Figures S1 and S2).

**Figure 2:**
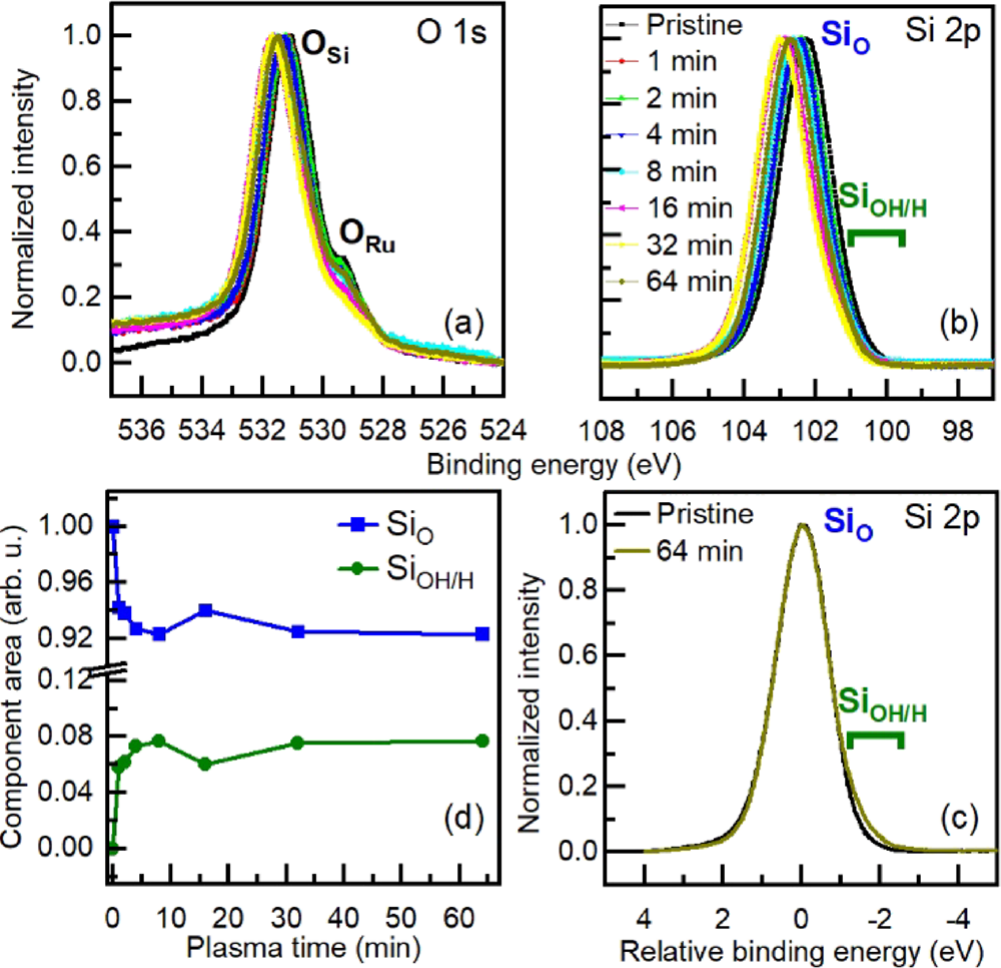
(a) O 1s and (b,c) Si 2p XPS lines for a crystalline SiO_2_ BL/Ru(0001) sample acquired after consecutive and incremental H-plasma exposures, as indicated. O 1s and Si 2p lines were collected with photon energies of 600 and 175 eV, respectively. (c) Superposition of Si 2p lines for the pristine SiO_2_ BL and 64 min plasma-treated sample. (d) Time evolution of the components’ areas obtained from the sample fitting of the Si 2p lines for the different silica samples. Fitting results of the complete time series are available in Section 4 of the Supporting Information.

Figure 2 shows O 1s and Si 2p photoemission spectra (XPS) at various stages of the treatment. A clear shift toward higher binding energies (BEs) of both the oxygen and silicon lines occurs with increasing plasma exposure time (Figure 2a,b), as well as a decrease in the contribution at ∼529 eV assigned to interfacial oxygen (Figure 2a). The same effect has been previously reported for the so-called O-poor state of the SiO_2_ BL/Ru(0001) system produced by two different approaches. First, interfacial O_Ru_ can be removed by thermal desorption through annealing of the SiO_2_/3O/Ru(0001) system at temperatures close to the onset for O-desorption (∼1150 K) under UHV conditions.^39,40^ In this case, the integrity of the silica film is heavily compromised due to local de-wetting of the film^25^ and only vitreous or mixed crystalline–vitreous phases are obtained because of the well-known phase transition of the bilayer.^26^ Second, O_Ru_ can also be removed by rather milder annealing treatments (typically 450–650 K) in a H_2_ atmosphere. This approach has the advantage of preserving the crystalline structure of the silica film. In this case, the O_Ru_ component is removed by reacting with H_ads_, thus generating water as a byproduct.^41^ Regardless of the mechanism followed, the origin of the shifts in silicon and oxygen lines is tracked down to the removal of the O–Ru surface dipoles. Desorption of the O_Ru_ species causes a change in the total effective surface dipole at the silica–ruthenium interface and in the Ru surface potential (work function), thus affecting the interaction of the BL and the ruthenium support. More details can be found elsewhere.^39,40^ It is important to note that reintercalation and readsorption of background gases can take place, especially for longer plasma treatments, therefore affecting the relative position of the Si and O core lines. However, this process does not interfere with the interaction of the silica film with the active species present in the plasma.

On the other hand, the small changes seen in the Ru 3d line follow the trend expected for the removal of O_Ru_ that were reported for the O-covered Ru(0001) surface without silica (see Figure S5). In this case, the removal of O_Ru_ eliminates the charge transfer from the Ru atoms, resulting in a more metallic character and therefore eliminating the contribution of components at higher BEs. Consequently, the contribution at lower BEs increases as the θ_O_ decreases. All these changes are in agreement with data reported for oxygen adsorption on bare Ru(0001).^42^

In addition to the removal of the O_Ru_, functionalization of the silica film is observed. A closer look into the evolution of the Si 2p line shape reveals the emergence of a new chemical state upon plasma treatment at lower BEs. Figure 2c shows the superimposed spectra (energy is re-scaled to compensate for the dipole effect) of the Si 2p lines collected for the pristine and the 64 min plasma-treated sample. It becomes clear from this direct comparison that the new chemical species formed during plasma exposure is/are responsible for the shoulder on the lower BE. The time dependence of the components’ intensity was monitored by applying a consistent peak fitting protocol of the Si 2p line. Details on the fitting procedure adopted and the individual results for the consecutive exposures can be found in the Supporting Information. Two components were used in the fitting: the one at a higher BE is ascribed to the typical Si–O species in the pristine SiO_2_ crystalline BL (bottom and top layer). The second component appearing at a lower BE is assigned to either Si–H or Si–OH formed by the interaction of Si–O–Si bridging bonds in the pristine silica BL with the reactive species present in the hydrogen plasma. It is worth mentioning at this point that negative BE shifts have been observed for OH groups on various silica samples in liquid media, with their respective chemical shifts being dependent on the pH of the liquid media due to deprotonation.^28^

The time dependence analysis of the (integral) intensity of the new component shown in Figure 2d indicates a sharp increase in its production at short exposure times, reaching saturation at approximately 4 min of plasma exposure. The saturation behavior suggests that there is a limit in the degree of functionalization of the BL, probably due to the stability of the new species created or the surface concentration of active sites/defects acting as anchoring points for the reactive H-species. For instance, it is known from previous STM experiments that even though the crystalline phase of the silica BL is expected to be a perfect system constituted by hexagonal channels formed by 6-member rings (either of O or Si atoms), in reality this phase presents a series of defects/domain boundaries that are defined by rings of smaller and larger sizes.^24^

**Figure 3:**
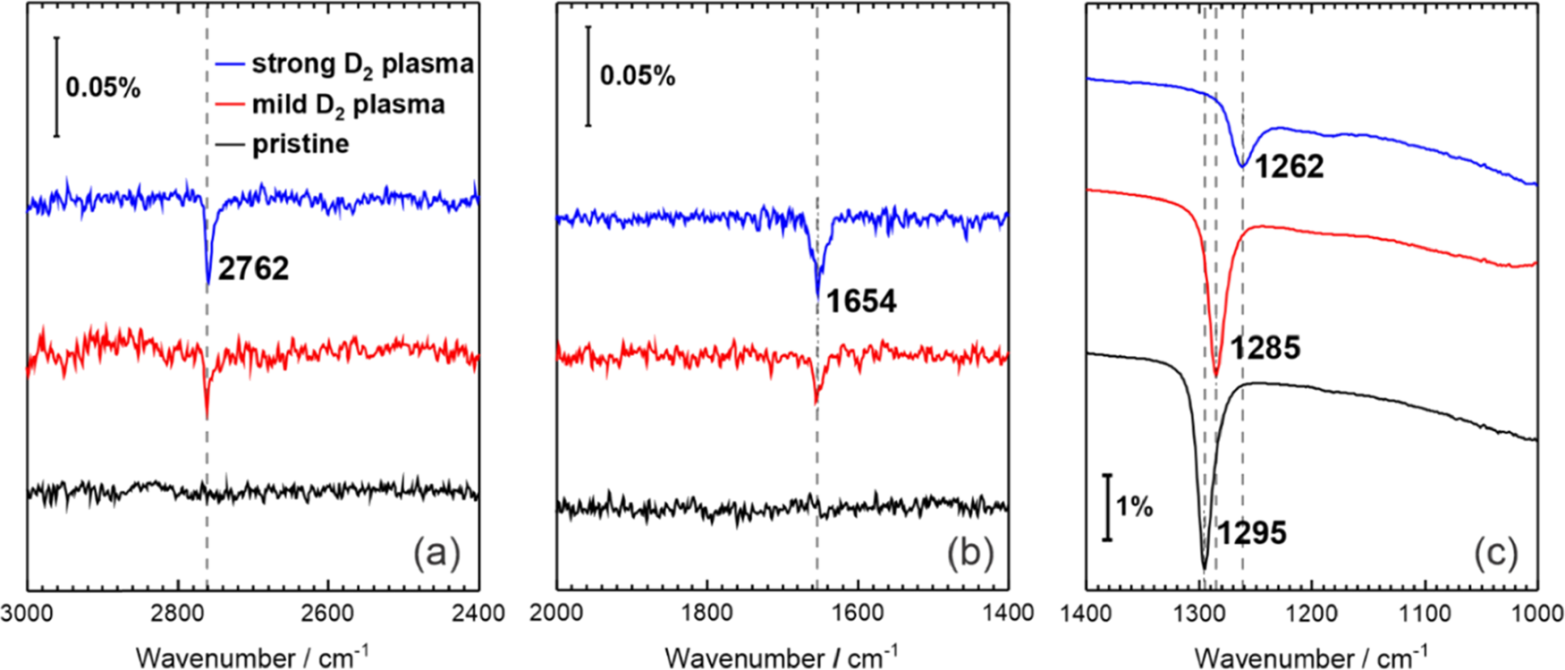
IRAS spectra of a crystalline SiO_2_ BL/Ru(0001) showing the (a) O–D, (b) Si–D, and (c) Si–O–Si vibration modes under different H-plasma treatment stages. The operating anode voltages were 400 V (mild) and 800 V (strong). Consecutive plasma exposure time was 20 min in both cases. The plasma source was operated with deuterium at a pressure of 1.2 × 10^–4^ mbar. The dashed lines indicate the positions of the absorption lines.

Complementary to the XPS data collected, we performed IRAS experiments in some selected samples. For all IRAS experiments, the plasma source was operated with deuterium instead of hydrogen, and only plasma conditions leading to a relatively high degree of functionalization of the crystalline silica BL were used. The two parameters tuned were the long plasma exposure time and the strength of the plasma source by means of higher anode voltages of the source operated in the ion mode. Figure 3 shows the IRAS spectra of different analyzed samples. The spectra of the pristine BL (black line) show a rather intense and well-defined band in the range 1295–1262 cm^–1^. The position of this band is in excellent agreement with the value reported for the Si–O–Si vibration of the bridging bonds within the two coupled layers^43^ and taken as the IRAS fingerprint of the BL polymorph. The lack of any other signal in the range of wavenumbers explored confirms the integrity of the film and the saturation of the Si–O–Si bonds.

On the other hand, two other bands appear at 2762 and 1654 cm^–1^ upon both plasma treatments. Because the intensity of both bands clearly increases with the strength of the plasma conditions (mild plasma vs strong plasma), we assign these signals to the −OD or −D groups, respectively.^30,44^ The degradation and shift of the band at 1295 cm^–1^ are consistent with the breaking of Si–O–Si bridging bonds upon functionalization. According to DFT, this band is associated with the in-phase displacement of the bridging oxygen atoms in the direction perpendicular to the surface, which leads to a strong alteration of the z-component of the dipole moment and hence a high infrared (IR) intensity. Disappearance of some of the bridging Si–O–Si bonds increases the weight of other atoms’ displacements in this mode, which lowers its intensity and shifts the frequency. A similar effect has also been observed and reported previously.^30^

DFT calculations were performed to identify the structure and geometry of functionalization of the BL (inter- vs intralayer). The fact that we observe single bands for both Si–D and O–D stretching modes suggests that any structure should result from the single addition of H_2_ or D_2_ molecules, thus leading to relatively isolated Si–H and Si–OH groups. Moreover, the comparison of the LEED patterns collected before and after plasma exposure (Figure 1) suggests that, to maintain the registry between the BL and the Ru(0001) support, the in-plane order must be largely preserved upon functionalization. Consequently, it seems reasonable to identify the interlayer Si–O–Si bonds as being more susceptible to functionalization, thus ensuring that the ring-like structure remains mostly unchanged and preserving the long-range order.

Within the single addition scenario, different structures were considered in our modeling. Considering the symmetry properties of the system and the possibility of inter- or intralayer functionalization, five different structures were investigated. All structures studied (single and double H_2_ addition) and their respective IRAS spectra can be found in the Supporting Information.

**Figure 4:**
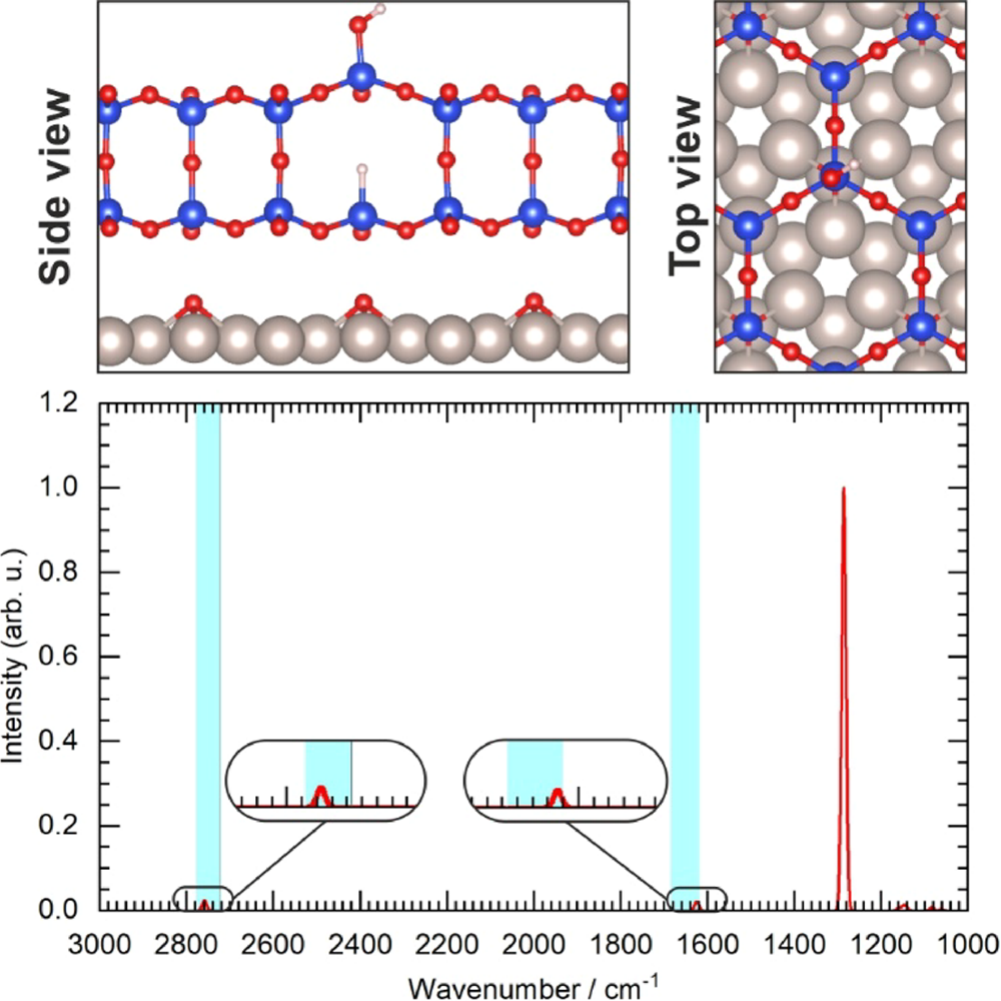
Top panel: atomic model of the functionalized silica BL showing the position of −D and −OD groups. Bottom panel: IRAS spectra calculated for the structures shown in the top panel. Semitransparent blue boxes indicate the regions where the vibration signals were experimentally observed.

Figure 4 shows the results obtained for the structure having the best correlation with the experimental dataset. An excellent agreement has been found for both Si–OD and Si–D bands resulting from the interlayer functionalization model, with the OD group located on the top layer and the SiD at the bottom of the SiO_2_ BL. It is important to note that the complementary structure with the OD and SiD positions exchanged renders a rather poor correlation with the O–D band, with a shift of 110 cm^–1^. The same shift has been found between O–D groups in the top and bottom layers of hydroxylated SiO_2_ BL/Ru(0001) and ascribed to the interaction of the hydroxyl group with the bridging oxygen atoms through hydrogen bonds.^30^ On the other hand, additions considering the intralayer functionalization are discarded based on the discrepancy of the obtained vibration frequencies for such structures of both O–H (O–D) and Si–H (Si–D) stretching modes.

The BE shift of the Si 2p line was calculated for all structures considered, and a negative shift is predicted for both functional groups, in good agreement with the experimental results (see Figures 2 and S6 of the Supporting Information). However, Si–H groups tend to exhibit slightly stronger chemical shifts in comparison with Si–OH species, in agreement with predicted chemical shifts in the Si 2p line of hydridospherosiloxane (H_8_Si_8_O_12_) clusters.^45^

Moreover, contrary to the single addition structures, our calculations anticipate site heterogeneity for the structures resulting from double addition (see Sections 5.1 and 5.2 of the Supporting Information) reflecting the local interaction of the functional groups via H-bonding. Nevertheless, we conclude that Si–H bonds exhibit stronger chemical shifts and larger heterogeneity in comparison with Si–OH bonds, thus echoing the fact that the electronegativity of H lies between that of Si and O and that hydrogen is more polarizable.

Similar results are obtained with a vitreous polymorph (see Sections 3 and 4 of the Supporting Information). However, two main aspects stand out from the comparison between the two polymorphs. First, the extent of O_Ru_ removal seems to differ among the polymorphs. In the case of the crystalline film, the observed BE shift for the Si 2p line is ∼0.4 eV smaller than that of the vitreous film (see Figure S8), thus suggesting a higher permeability toward hydrogen for the latter. In this sense, the larger pores (7–9 member rings) in the vitreous film may overcompensate the lower permeability of smaller ring sizes (4–5 members). This agrees with the reported higher reaction front velocities observed for the water formation reaction under a vitreous BL.^38,41^

Second, saturation levels of OH/H groups clearly differ when between polymorphs. While in the case of the crystalline film, a saturation is reached at 8%, the vitreous film yields a lower saturation value of about 1%. Considering the periodic nature of the crystalline structure, we can estimate from the XPS data that a maximum surface concentration of 1.32 nm^–2^ is reached at the saturation level, a more than three times greater functionalization in comparison with the electron bombardment route previously reported.^15^

A simple model depicting the possible active sites for functionalization is presented in the Supporting Information (see Figure S23). For the crystalline polymorph, the saturation limit can be rationalized based on the following argument. If the formation of a pair of Si–OH and Si–H groups blocks the three involved 6-member rings for further hydroxylation, then a  superstructure with respect to the Ru(0001) lattice is anticipated. This superstructure contains 12 Si atoms from the two layers of the silica film, with only one OH/H pair per unit cell. Therefore, the ratio of 1/12 (0.083) defines the theoretical limit for functionalization of the crystalline film, in excellent agreement with the value derived from our XPS results presented in Figure 2.

The origin of the saturation level difference may be twofold. First, the vitreous BL offers (locally) larger pores for permeation in comparison with the crystalline BL. Therefore, the impact frequency of activated H_2_ molecules with the film can be locally lower. In areas where the pores are larger, hydrogen permeation through the silica film has a much lower penetration energy barrier,^46^ and therefore, interaction of plasma species with the BL would be only a short-lived event. Second, because the saturation limits differ significantly, one may assume that there must be an intrinsic difference in how the different polymorphs cope with defect formation. Recently, Gura et al.^47^ studied how the structure of a SiO_2_ BL films can be locally affected by the presence of steps on the Ru support. Interestingly, deviations in the ring–ring and Si–Si interatomic distances were observed in the proximity of step edges based on DFT simulations. These findings clearly indicate that, even though the silica BL interacts with the substrate only via (weak) dispersive forces, the local structure on the Ru surface can induce strain in the silica film. The authors reported on the effect of monoatomic steps, but we anticipate that this effect must be even more pronounced in the vicinity of the step bunches, where height variations are more pronounced. It is evident from the LEEM images in Figure 1a (and S1–S3) that step bunches are rather common at mesoscopic scales in this system (bright and dark lines in LEEM). Therefore, we propose that areas in the silica film initially submitted to local stress (tensile or compressive) are more prone to react with the active components of the plasma and finally result in the formation of stable Si–OH and Si–H bonds.

### Direct Applications of a Functionalized SiO_2_ BL

The heterolytic splitting of molecular hydrogen on oxide surfaces has been extensively discussed in the literature^48^ for oxides such as MgO,^49^ TiO_2_,^50^ CeO_2_,^51^ Al_2_O_3_,^52^ and ZnO.^53^ In line with these reports, heterolytic dissociation of plasma-activated hydrogen also occurs on a silica BL. However, differently from the routes described in the literature, H_2_ splitting involves charged species (H_2_^+^). A complementary theoretical study aiming at a complete description of the mechanistic aspects is necessary to describe how charge is redistributed upon splitting.

On the other hand, the interaction of molecules with active sites in solid oxides is a topic that has been addressed extensively among the catalysis community. Particularly, in the case of zeolite-based materials, the interaction of adsorbate molecules with acid sites provides a way of determining the reactivity and availability of active sites for a variety of reactions.^54^

Particularly interesting is the use of the SiO_2_ BL/Ru(0001) model to study confinement effects on the kinetics of chemical reactions.^38,55^ Recent microkinetic studies indicate that the permeability of the silica BL can drastically affect the global kinetics of the water formation in confinement.^38^ For instance, the presence of the silica lid introduces additional transition states in the H_2_ adsorption and H_2_O desorption steps. For H_2_O molecules in particular, molecules can be stabilized inside the SiO_2_ cage by H-bonds with the OH groups, thus affecting the transient water coverage on ruthenium. Consequently, paths like disproportionation/comproportionation can then become relevant and affect the overall observed kinetics.

On the other hand, a new playground emerges for the field of SACs when using the functionalized silica as a 2D support. Among many important parameters affecting the reactivity and selectivity of SACs, strong interaction of the active phase (single atom) with the support and its dispersion seem to be highly correlated.^56^ A strong interaction of the TM atom can not only determine/modify the electronic properties of the active site but also avoid that sintering degrades the long-term stability of the catalyst.^57^ In this sense, the functionalized SiO_2_ BL system constitutes an suitable model system to study these properties.

Because OH and H groups are in well-defined positions, active centers anchored in the structure SiO_2_/Ru(0001) BL structure would result in a good dispersion, provided that the metal ad-atoms interact strongly with OH and/or Si(H). In this sense, the affinity of TMs for oxygen or silicon can determine on which side of the BL the active phase will bind. Moreover, atoms binding to the bottom layer through the SiH group can benefit from confinement effects arising from the presence of the ruthenium support. It is important to highlight at this point that even though our work foresees a saturation limit for the functional groups, it is not clear if the structure adopted by the H/OH pair is entirely periodic. However, we anticipate that the average distances of about 0.9 nm between neighboring anchoring sites would ensure that the single atom active sites are well-distributed on the silica support. Finally, if two different atoms are anchored simultaneously (top layer: Si–O–TM_1_; bottom layer: Si–TM_2_), the two active sites are only a few angstroms apart, thus defining a model system for studying fundamental concepts on coupled reactions in tandem catalysts where the selectivity/reactivity of a chemical reaction is stirred by the combination of multiple active sites working in a synergic manner.^58^

## Conclusions

We demonstrate that exposing different silica BL polymorphs (crystalline and vitreous) to H-plasma results in the functionalization of the BL with formation of neighboring-OH and -H functional groups without any change in the 2D structure of the films. Comparison of the experimental XPS and IRAS with theoretically derived counterparts reveals that functionalization takes place through the heterolytic dissociation of the Si–O–Si bond bridging the contiguous layers. Our study concludes that, while the Si–H group is located at the bottom layer (thus remaining inside the silica cage), the OH group binds to the Si atom located in the topmost layer. The functionalization process appears to follow the same mechanism in different polymorphs of the BL. By comparing the response of the vitreous and crystalline polymorphs, we identify different saturation levels for the functionalization, with the vitreous film being the one showing a lower reactivity toward plasma functionalization (1% vs 8% functionalization). The difference in reactivity can be traced down to the difference in the local structure of the polymorphs and their capability of stress dissipation.

Finally, the fact that the functional groups created upon plasma treatment are located in well-defined positions of the silica BL projects the application of these chemically modified 2D SiO_2_ films as model support systems. We envision their future use as conducting supports in electrochemistry applications that favor the stabilization of SACS and small clusters. Thus, they will constitute an excellent platform for the study of fundamental steric/geometric and electronic effects of multicomponent single atom/small cluster catalysts. Moreover, effects such as interatomic distances between active sites and confinement of the active centers can be addressed in a more controlled and reproducible manner with this new model support.

## Experimental Section

The experiments were carried out in the Spectro-Microscopy with Aberration correction for many Relevant Techniques (SMART) microscope operating at the UE49-PGM beam line of the synchrotron light source BESSY II of the Helmholtz Centre Berlin (HZB). This aberration corrected and energy filtered LEEM/PEEM instrument combines microscopy (LEEM/XPEEM), diffraction (μ-LEED), and spectroscopy (μ-XPS) techniques for comprehensive characterization. The base pressure of the system is 10^–10^ mbar, but operation is possible at pressures up to 10^–5^ mbar of reactive gases in a temperature range between 150 and 1500 K.^59^

The Ru(0001) single crystal was prepared by cycles of Ar^+^ sputtering at room temperature and annealing in oxygen at 1170 K. Subsequent annealing steps at 1520 K in UHV rendered flat Ru(001) surfaces with a few-hundred nanometer wide terraces. Cleaning cycles were repeated until no contamination could be detected by XPS, with terraces a few 100 nm wide and a sharp (1 × 1) LEED pattern. Sample temperature was measured either using a W26%Re/W5%Re thermocouple or using a pyrometer (IMPAC IGA 140) with an absolute accuracy of ∼10 K. Gases were dosed either directly into the experimental chamber (oxygen −99.999%) or to the plasma (hydrogen (99.999%) differentially pumped chamber. Silicon was sublimated onto a 3O-Ru(0001) surface from a 4 mm thick rod (99.999%) using a commercial evaporator (Omicron EFM3) under a grazing incidence of 20°.

Two types of SiO_2_ BL polymorphs were used in all experiments, namely, crystalline and vitreous. Silica films were produced, following well-defined protocols that can be found elsewhere.^25^ However, we offer in the following a short description on the steps followed. First, a so-called 3O layer (θ_O_ = 0.75 ML) is formed on the Ru(001) surface by exposing the ruthenium crystal to 1 × 10^–6^ mbar O_2_ at 1179 K in the last cleaning step. In a subsequent step, the necessary amount of Si is deposited at room temperature in a background O_2_ atmosphere of 2 × 10^–7^ mbar. In the last step, the oxidation and ordering of the SiO_2_ BL are performed by annealing the SiO_*x*_/Ru(001) system in 1 × 10^–6^ mbar O_2_. The choice of the annealing temperature ultimately determines the type of polymorph (crystalline or vitreous) obtained at the end of the process.

However, it is important to mention that as a result of the preparation step, both SiO_2_/Ru(0001) polymorphs contain an intercalated O layer adsorbed on Ru with estimated coverage of ∼0.75 ML that of the well-known 3O phase in a (2 × 2) atomic arrangement.^60^ This state is known as the O-rich phase and corresponds to the initial stage of our plasma exposure experiments.

Plasma treatments were conducted by exposing the SiO_2_/Ru(0001) sample to H_2_ plasma generated by a commercial UHV-compatible plasma source (MPS-ECR, SPECS GmbH). Plasma conditions were always kept constant, using the exposure time as the only variable, unless specifically stated. The plasma source was operated at p(H_2_) = 1 × 10^–4^ mbar in the hybrid mode (ion + atoms) with an ion energy of 400 eV, set by the anode voltage. Typical values of magnetron current/voltage of 15 mA/3.7 kV and extractor current/voltage of −6 μA/–200 V were used. The sample was exposed to the plasma beam only after stable conditions were achieved. In addition to this so-called mild treatment, a strong treatment was performed at 800 eV for an accumulative time of 40 min in the IRAS setup, to determine the stability of the silica film.

IRAS experiments were conducted in a separate chamber equipped with LEED, XPS, and IRAS. The IRA-spectra were recorded with a Bruker IFS 66v spectrometer using p-polarized light at an 84° grazing angle of incidence with a resolution of 4 cm^–1^. The samples were treated with a microwave plasma source (from Oxford Scientific) using D_2_ (99.999% purity, 99.8% isotope, Linde).

## Computational Methods

Structure optimizations, as well as evaluation of IR spectra and BE shifts were performed at the DFT level, using the PBE functional^61^ and an additional dispersion correction D2.^62^ Calculations were carried out, using the plane-wave code VASP,^63^ employing the projector-augmented wave method to represent core states. The first Brillouin zone was sampled using a weighted, uniform 4 × 4 × 1 k-point grid.^64^ The plane wave energy cut-off was chosen to be EPW = 400 eV. To accelerate SCF convergence, a Methfessel–Paxton^65^ type smearing with a width of 0.05 eV was utilized. The convergence thresholds in the structure optimizations were 5 × 10^–3^ eV Å^–1^ for forces and 1 × 10^–5^ eV for the energies. BE shifts were estimated using the initial state approximation.^66^

For the initial structure optimization, we used a five-layer Ru-slab with the two bottom layers fixed at the bulk position. The BE shifts were then calculated using the optimized structures. For the calculation of IR spectra, the Ru-slab was reduced to three layers and subsequently reoptimized with only the bottom layer fixed at the bulk position. The displacements of the Ru atoms were excluded from the computed Hessian matrix. A dipole correction was added, to compensate for spurious interactions due to the dipole moment in the direction orthogonal to the surface. The Hessian matrix and the dipole moment changes were evaluated, using a central finite-difference scheme. For comparison to experimental IR spectra, the calculated intensities include only the component orthogonal to the surface of the respective Born charges.

To correct for systematic errors within the harmonic DFT calculations, the frequencies of the Si–O–Si modes were scaled by a factor of *f*(SiOSi) = 1.0341,^43^ the O–D modes *f*(OD) = 0.9951,^30^ while the Si–D mode frequencies were not scaled, *f*(SiD) = 1. The latter was based on the fact that the harmonic PBE-D2 frequencies for the SiD_4_ molecule virtually reproduce the experimental values (PBE-D: 1589.5, 1589.3, and 1589.2 cm^–1^; experiment:^67^ 1592.7, 1589.2, and 1584.7 cm^–1^).

## Supporting Information Available

Low energy electron microscopy image time series of all analyzed SiO_2_ films; low energy electron diffraction patterns of all analyzed SiO_2_ films; evolution of Ru 3d core lines during the plasma exposure of a crystalline SiO_2_ BL; fitting results of the Si 2p line across the different plasma treatments; detailed description of the different models used for the DFT calculations; theoretical prediction of binding energy shift expected for Si–D and Si–OD components under the Si 2p core line; predicted IRAS spectra of the different models considered for a functionalized SiO_2_ BL; relative total energies of all functionalized SiO_2_ model structures; and model describing the possible active sites for functionalization on a crystalline SiO_2_ BL (PDF)
