# Supplementary material for: Plasma Functionalization of Silica Bilayer Polymorphs
Source: ACS Appl Mater Interfaces. 2022 Oct 18;14(43):48609–18. doi: 10.1021/acsami.2c11491 (PMC9634693; doi:10.1021/acsami.2c11491)
Supplement: Supplementary file 1 — am2c11491_si_001.pdf [file am2c11491_si_001.pdf]

Supporting Information

Plasma functionalization of silica bilayer polymorphs

Mauricio J. Prieto*[a], Thomas Mullan[b], Weiming Wan[a], Liviu C. Tănase[a], Lucas de Souza Caldas[a], Shamil Shaikhutdinov[a], Joachim Sauer[b], Denis Usvyat**[b], Thomas Schmidt[a] and Beatriz Roldan Cuenya[a]

[a]*Department of Interface Science, Fritz-Haber-Institut der Max-Planck-Gesellschaft, Faradayweg 4-6, 14195 Berlin, Germany.*

[b]*Institut für Chemie, Humboldt-Universität zu Berlin, Unter den Linden 6, 10099 Berlin, Germany.*

*prieto@fhi-berlin.mpg.de

**denis.usvyat@hu-berlin.de

Contents

1. Low energy electron microscopy and low energy electron diffraction observations S-3

2. X-ray photoemission spectra S-5

3. Density functional theory calculations S-8

3.1 Geometries S-8

3.2 XPS binding energy shifts S-13

3.2.1 Theory S-13

3.2.2 Results S-13

3.3 IR Spectra S-16

3.3.1 Pristine bilayer S-16

5.3.2 Defective bilayer S-16

3.3.3 Bond-saturating single D_2 type defects S-17

3.3.4 Vicinal I type defects S-18

3.3.5 Geminal type defects S-19

3.3.6 Vicinal II type defects S-20

3.4. Relative energies S-20

4. Kinetic analysis of the hydroxylation process S-21

5. References S-23

1. Low energy electron microscopy and low energy electron diffraction observations

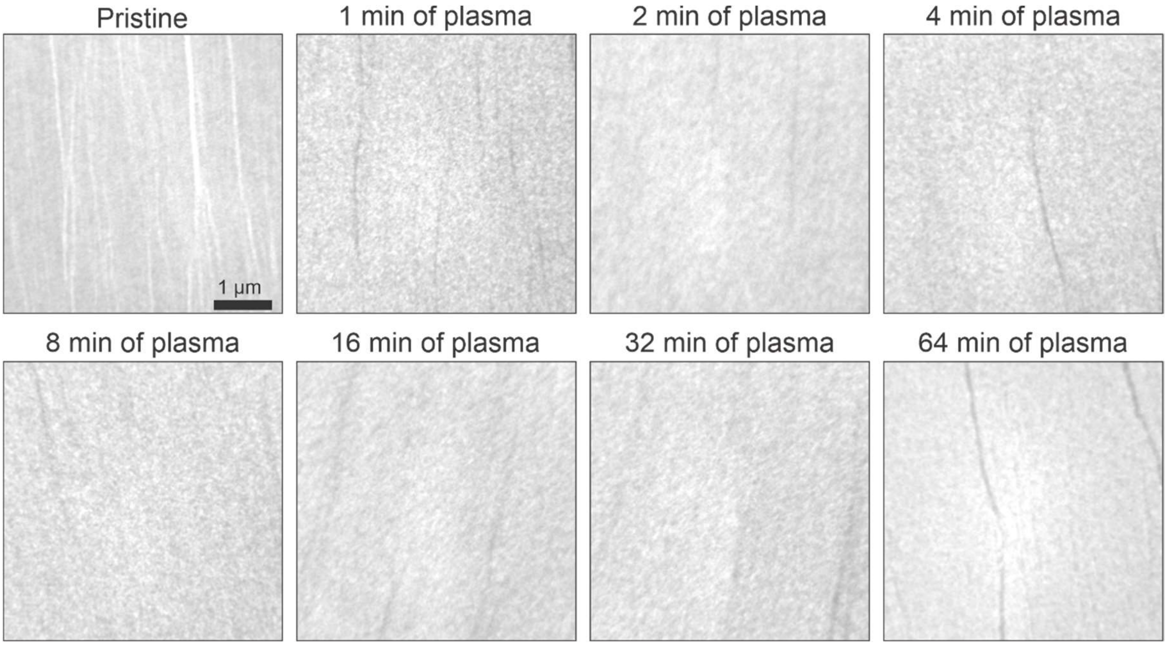

Figure S1. *LEEM images taken after consecutive and accumulative exposure of the crystalline SiO_2 bilayer polymorph to H-plasma, as indicated. Total exposure times vary between 1- and 64-min. Kinetic energy is 42 eV in all images.*

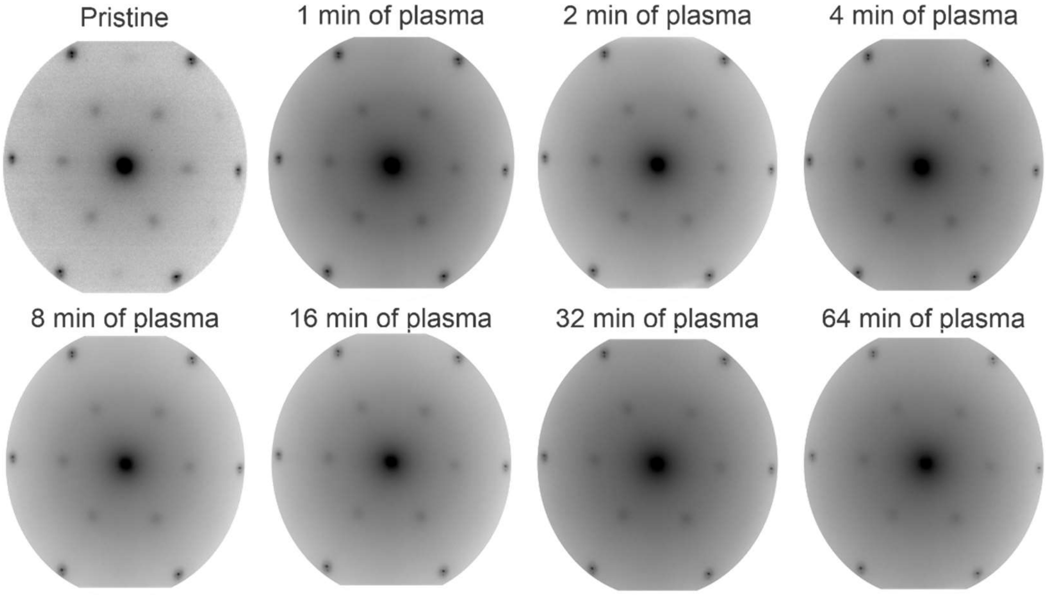

Figure S2. *LEED patterns taken after consecutive and accumulative exposure of the crystalline SiO_2 bilayer polymorphs to H-plasma, as indicated. Total exposure times vary between 1- and 64-min. Kinetic energy is 42 eV in all images.*

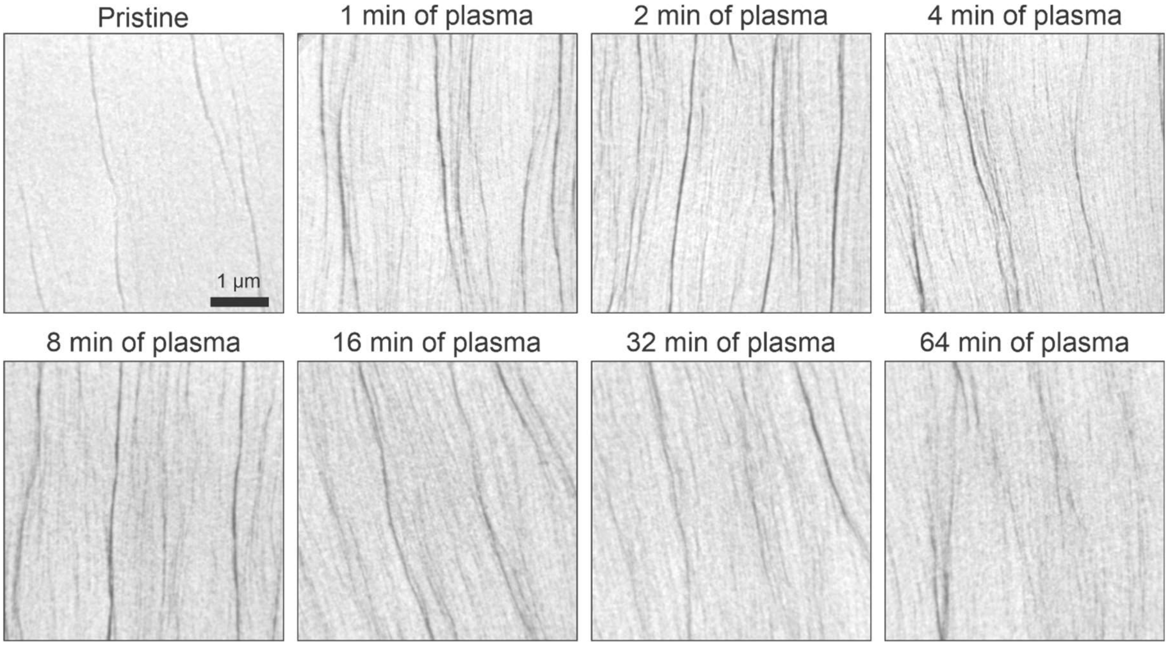

Figure S3. *LEEM images taken after consecutive and accumulative exposure of the vitreous* SiO_2 *bilayer polymorph to H-plasma, as indicated. Total exposure times vary between 1- and 64-min. Kinetic energy is 42 eV in all images.*

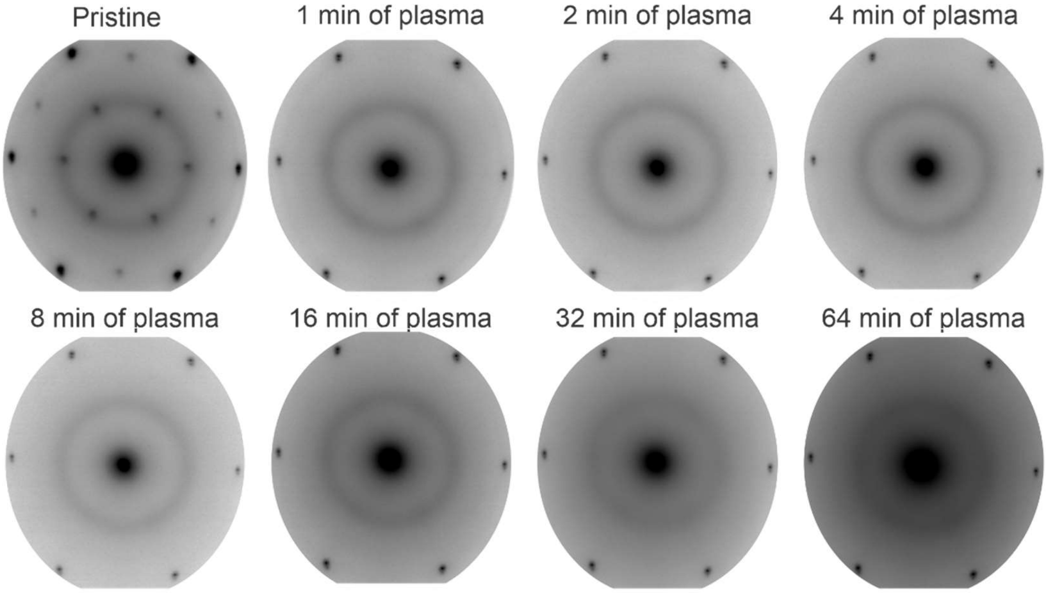

Figure S4. *LEED patterns taken after consecutive and accumulative exposure of the crystalline* SiO_2 *bilayer polymorphs to H-plasma, as indicated. Total exposure times vary between 1- and 64-min. Kinetic energy is 42 eV in all images.*

2. X-ray photoemission spectra

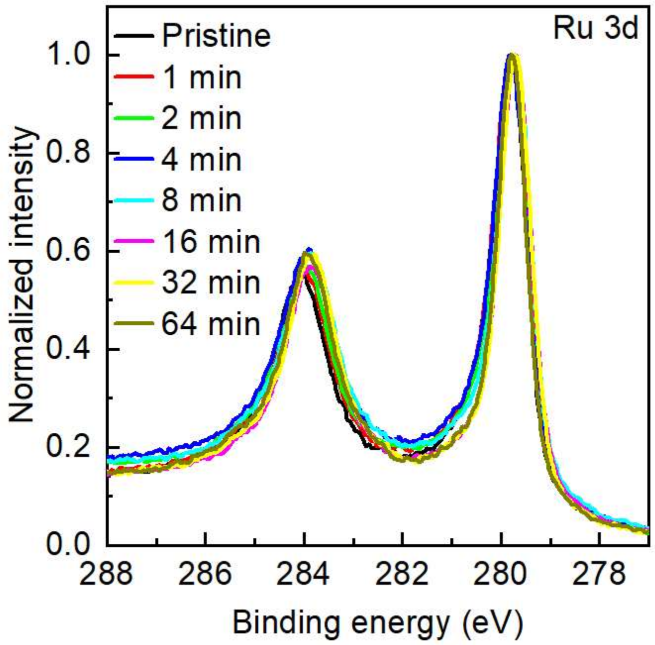

Figure S5. *Ru 3d photoemission line for a crystalline SiO_2 BL/Ru(0001) sample acquired after consecutive and incremental H-plasma exposures, as indicated in the legend. Photoemission line was collected with a photon energy of 360 eV.*

Fitting of all spectra was performed using a least squares curve-fitting protocol available in the WinSpec software. All lines were fitted using a Lorentzian line shape, unless stated otherwise. For the core-line analysis, a Shirley function was used to account for the background. A Gaussian broadening accounting for the instrumental contribution to the full width at half maximum (FWHM) and the line shape was estimated in a first step for the pristine sample and then maintained constant for all subsequent fittings corresponding to the different plasma exposure times. A doublet separation (spin-orbit splitting) of (0.63±0.02) eV and an intensity ratio of (1.96±0.05) were used for the Si $2p_{1/2}$ and $2p_{3/2}$ lines. The results obtained were validated by comparing the obtained FWHM of the different components to those reported in the literature.[1]

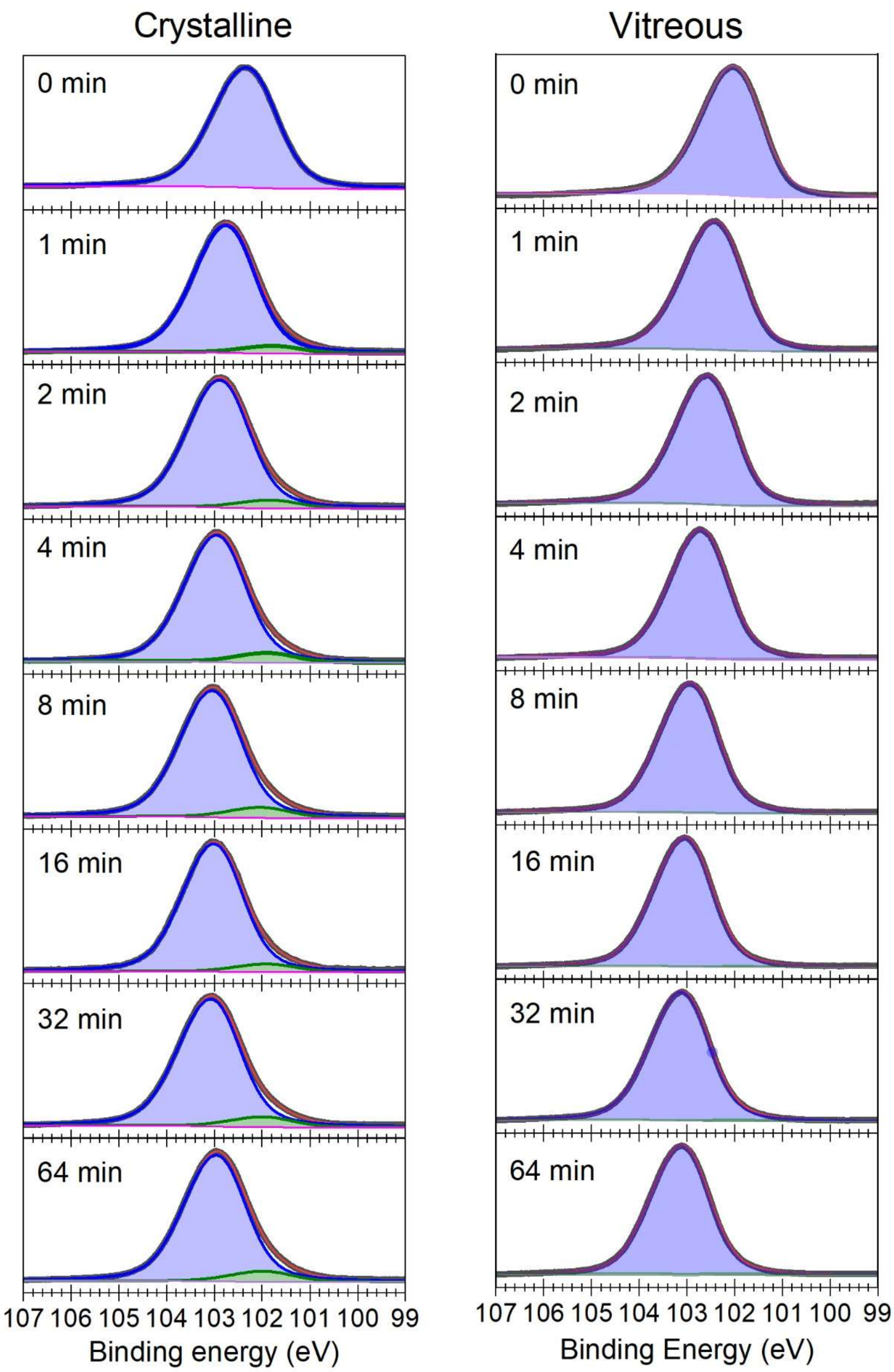

Figure S6. *Fitting results of the Si 2p photoemission spectra of the subsequently H_2-plasma treated crystalline and vitreous SiO_2 bilayer samples, as indicated.*

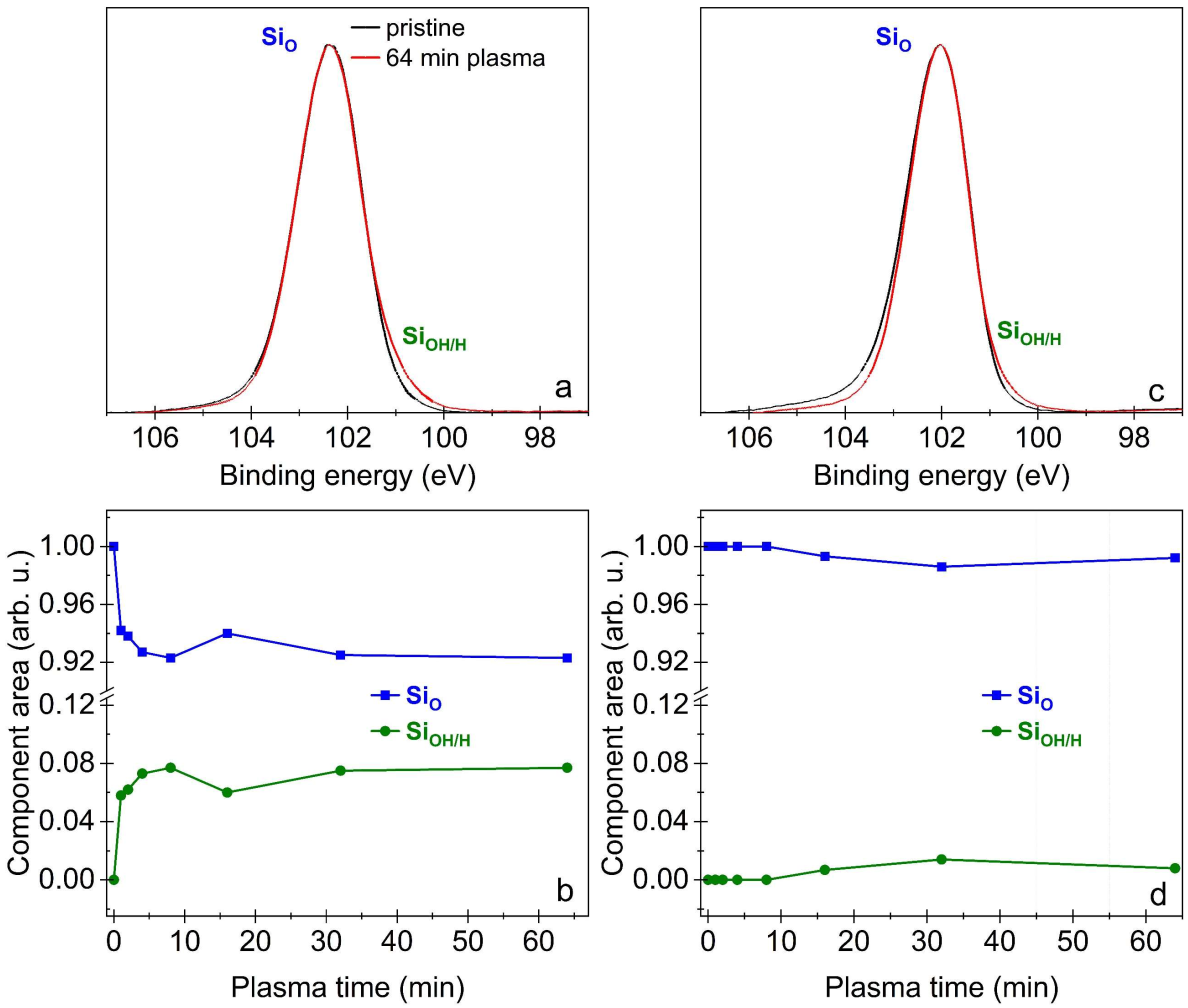

Figure S7. *Superposition of Si 2p lines (a,c) of the pristine and 64 min plasma treated SiO_2 BL, as indicated. (b,d) Areas of the components extracted from the deconvolution of the Si 2p line at different exposures. (a, b) correspond to the crystalline SiO_2 BL, while (c, d) correspond to the vitreous polymorph.*

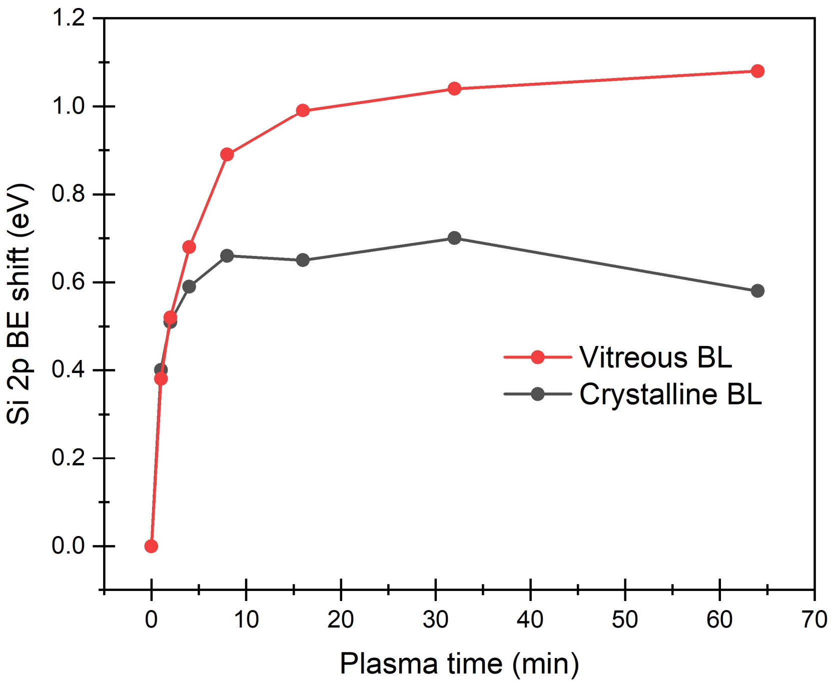

Figure S8. *Binding energy shifts observed for the Si 2p line as a function of H-plasma exposure time. The shifts were calculated from the spectra shown in Fig. S5.*

3. Density functional theory calculations

3.1 Geometries

The system under study is a repeated slab, consisting of five layers of 16 ruthenium atoms terminated with a (0001) surface, covered by chemically adsorbed oxygen atoms (4 oxygen atoms per unit cell, corresponding to a 1O-coverage in the nomenclature of reference[2]). A pristine silica bilayer is bound to the Ru-substrate on one side by van der Waals forces, while some (potential) defects may form covalent bridges to the surface (vide infra). The pristine bilayer is shown in Figure S9.

In this study we focus on pure hydrogenation defects. Experimentally, both deuterium and hydrogen are used for the functionalization of the bilayer, depending on the experiment. For the calculation of the IR spectra, all hydrogen atoms were replaced by deuterium atoms in the functionalized structure.

The first set of geometries considered results from the hydrogenation of a single Si-O-Si bond in the bilayer, consequently both hydrogen atoms must be located on neighbouring atoms. All considered structures are shown in Fig. S10. We refer to this set of structures as *bond-saturating* single D_2 type defects.

A second set of structures was obtained by adapting those in Ref.[3] In this study, the authors focused on defects of the silica bilayer that can be formed by the addition of two water molecules instead of hydrogen molecules. Consequently, we adapted the structures by replacing two OH groups with hydrogen atoms. Within this study, three types of structures were distinguished:

- vicinal I: here all defects (two OH groups and two H atoms) are evenly distributed amongst four directly neighbouring Si atoms. The structures are shown in Fig. S11

- geminal: here two defects are located on the same Si atom, while the other two are distributed amongst the remain two neighbouring Si atoms. This is equivalent two cleaving two Si-O-Si bonds for the same Si atom. We refer to these two sets (vicinal I and geminal) as *double* D_2 type defects. Representations of the structures are shown in Fig. S12.

- vicinal II: This set of structures is also adapted from Ref. [3], however in contrast to the other two, these structures feature covalent Si-Ru bonds between the bilayer and the metal substrate, in addition to two H atom defects in the top layer. Since this is equivalent to reacting with a single H_2 molecule as for the very first set mentioned, we also refer to these as under single hydrogen type defects. The two corresponding structures are shown in Fig. S13.

All obtained trial defect structures were re-optimized. Note that the numbers used in the nomenclature – 1 and 2 – refer to structural isomers of the same type, that include different hydrogen bonding motifs between hydroxyl groups and the interlayer oxygen atoms of the silica bilayer.

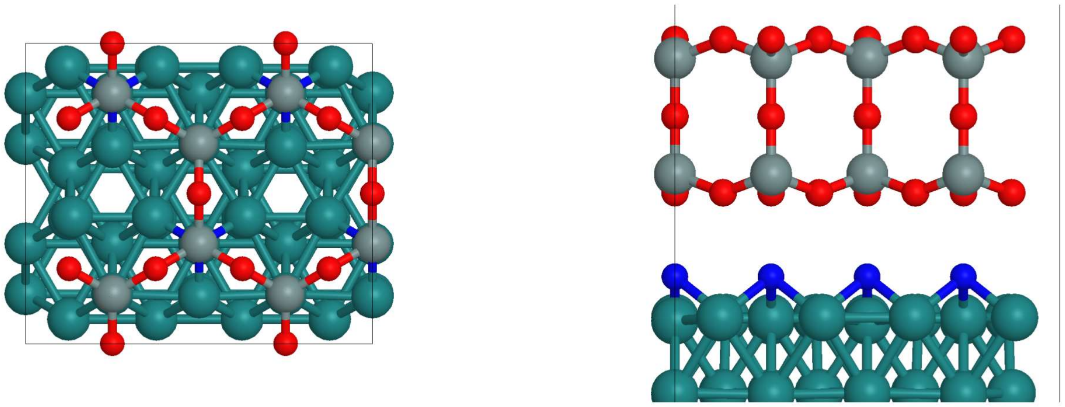

Figure S9. *Top (left) and side (right) view of the pristine bilayer model. Surface bound oxygens have been coloured blue for distinction from the bilayer. Green and grey balls represent Ru and Si atoms, respectively.*

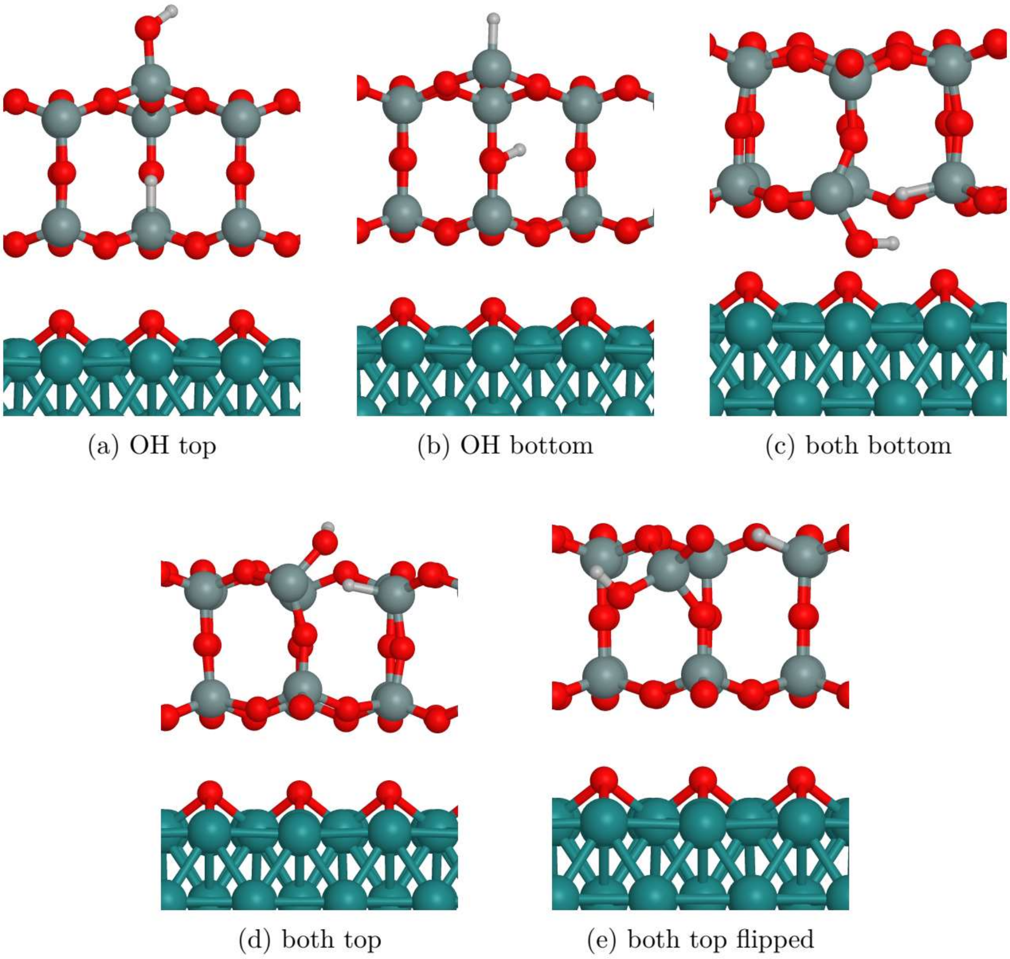

Figure S10. *Different bilayer configurations for bond-saturating single D_2 type geometries*

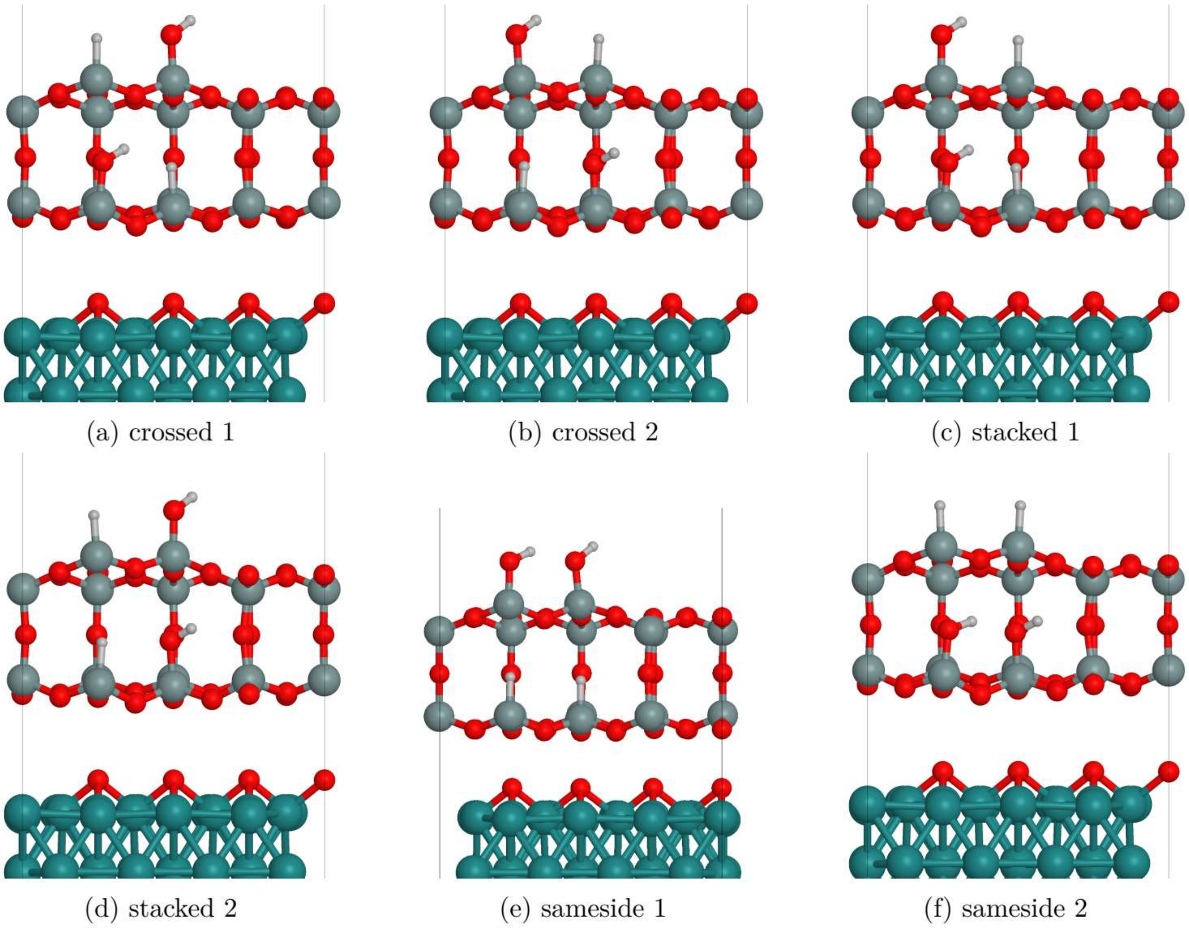
(a) crossed 1 (b) crossed 2 (c) stacked 1

(d) stacked 2 (e) sameside 1 (f) sameside 2

Figure S11. *Different bilayer configurations for vicinal I type geometries. The number – 1 or 2 – labels different permutations of the OD groups.*

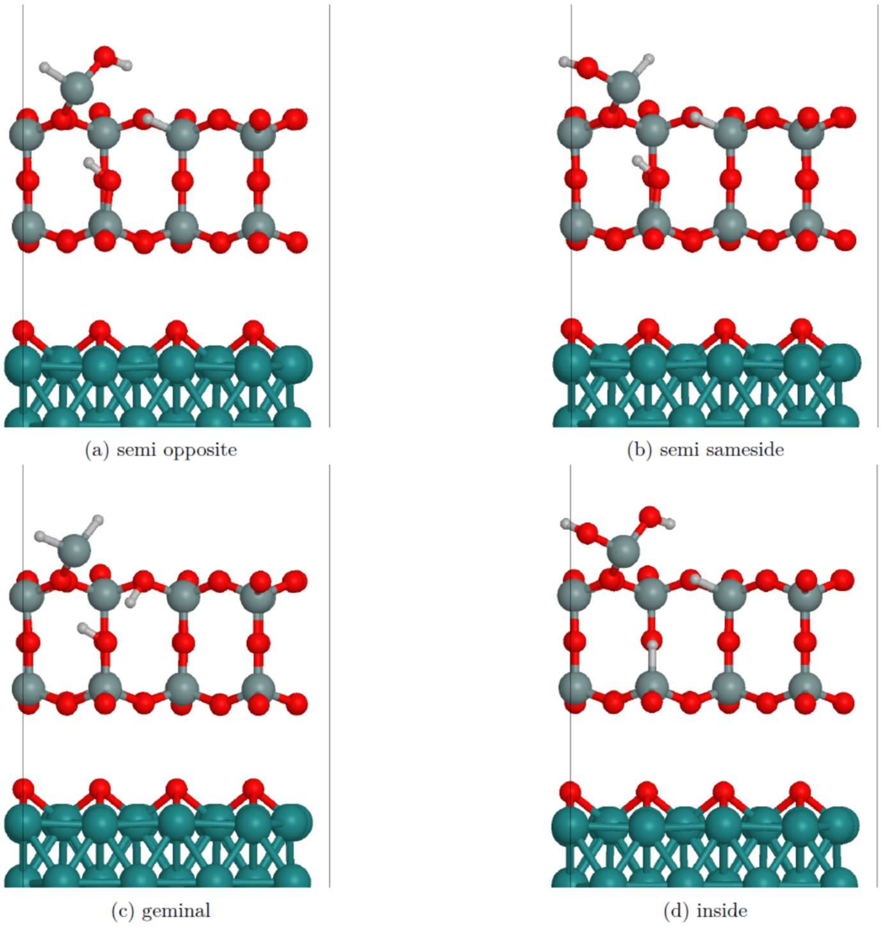

Figure S12. *Different bilayer configurations for geminal type geometries.*

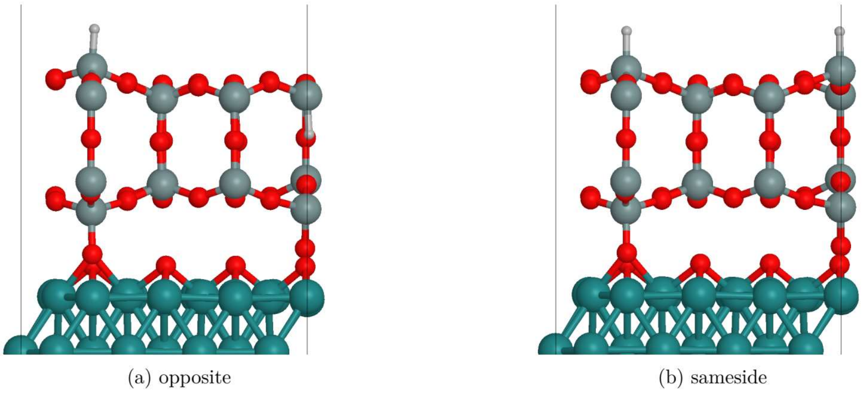

Figure S13. *Different bilayer configurations for vicinal II type geometries.*

3.2 XPS binding energy shifts

3.2.1 Theory

X-Ray photoelectron spectroscopy (XPS) is an established tool to probe atomic species and their chemical environment in a sample. It exploits the fact that tightly bound core electrons, although not directly participating in chemical bonding, are strongly influenced by the effective potential from the valence electrons, which in turn are strongly affected by local changes in geometry and composition. By measuring the ionization potentials (IPs) for the core electron ionization and the shifts in the IPs in a reaction, one can extract information on changes in the local chemical environments of these atoms.

The physical quantity of interest is the binding energy (BE) of an electron, which is obtained as the difference between the ionized and the ground state energy. In a periodic context however, a direct calculation of an ionized system is difficult, especially when handling core states in the context of plane wave basis sets. The simplest approach to cope with this problem is to consider the ionization potential within Koopmans' theorem, i.e. using orbital energies, resulting in the so-called initial state approximation. Core states are then represented using pseudo-orbitals formed in the projector augmented wave (PAW) method, as implemented in VASP.[4]

Within this approximation, the absolute BEs are not sufficiently accurate but, due to error compensation, the relative BE shifts are of reasonable quality, in particular for the SiO_2 bilayer.[5]

3.2.2 Results

The obtained BE shift values (Figs. S14 to S17) for the atoms under consideration exhibit a uniformly negative trend in the range between 0 and -0.8 eV. On close examination, one can find a dependence of the shift value on the H-bonding configuration in the structure. For the single H_2 type structures, there is one outlier for the both-bottom type geometry (Fig. S13), likely resulting from the strong interaction of the OH group with the metal interface. In all cases, the hydrogenated silicon atoms show a slightly stronger shift than the hydroxylated species. Although these results are in line with the experimental shifts, they do not allow the identification of any particular candidate structure. In order to characterize distinct features for each structure, we also calculated the IR spectra in order to compare them to the experimental spectra. For all other structures representing the double addition of H_2 (D_2) molecules, the same trend is observed, with some heterogeneity in the calculated BE shifts reflecting the local interaction of the functional groups via H-bonding.

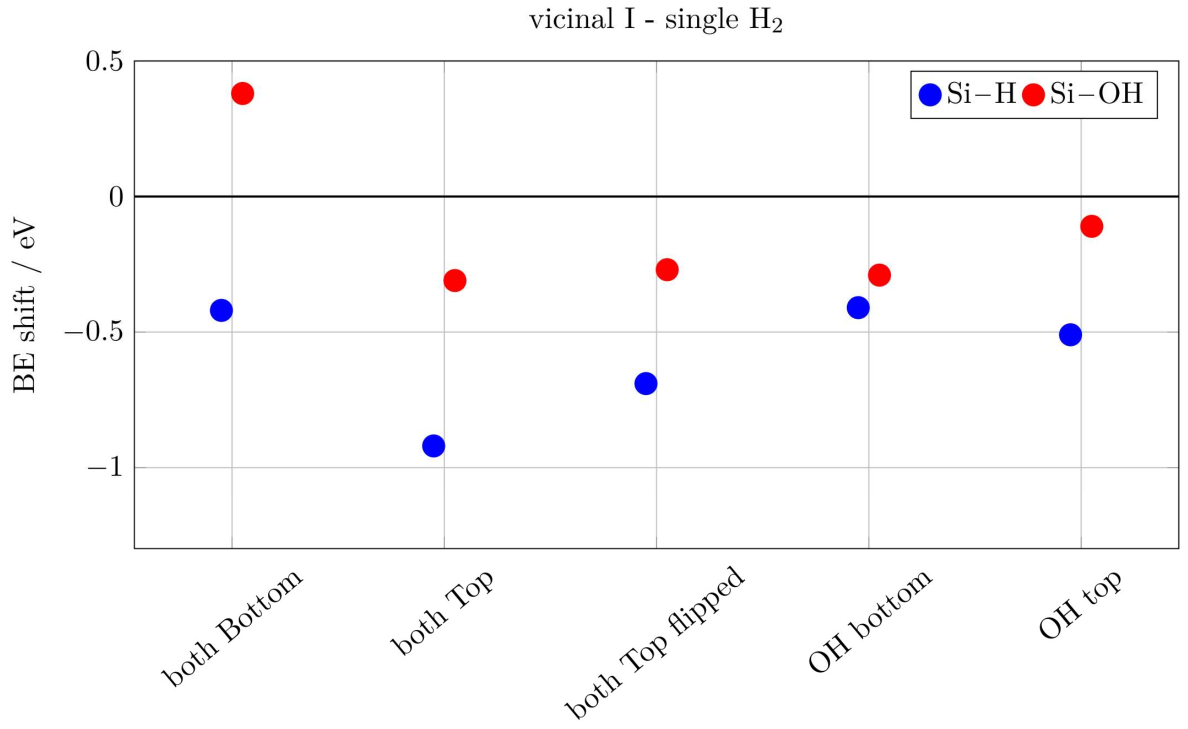

Figure S14. *Bond-saturating single H_2 Si 2p BE shift values for selected atoms.*

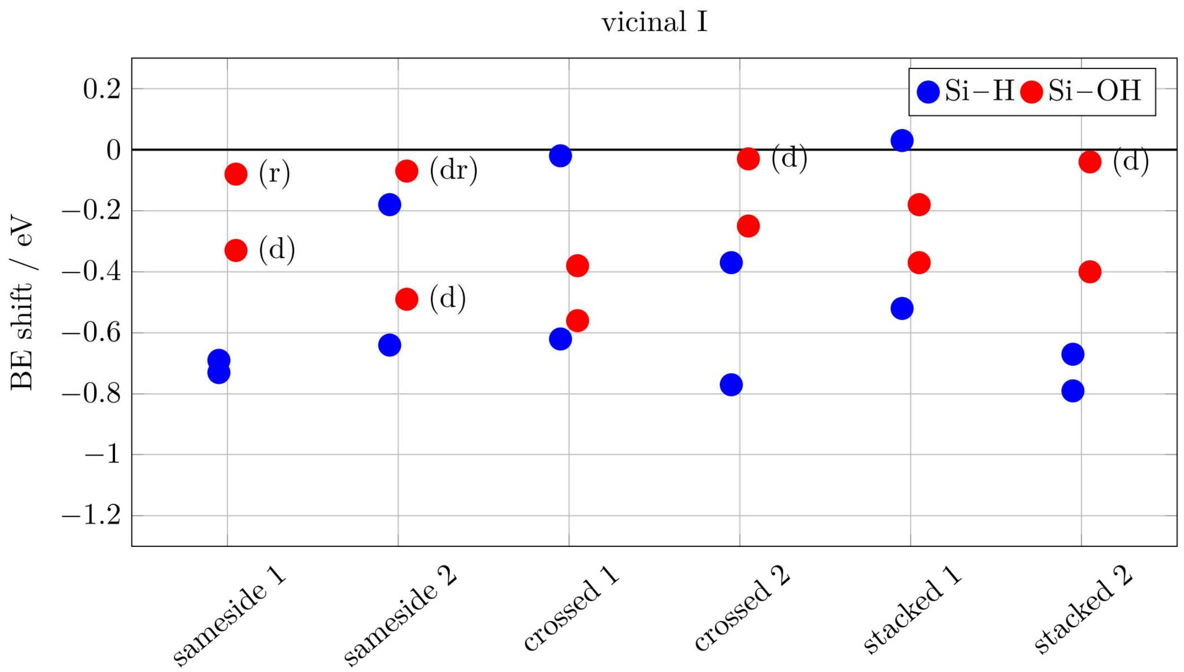

Figure S15. *Bond-saturating double addition of H_2. Vicinal I Si 2p BE shift values for selected atoms. The markers (d) and (r) label donating and receiving type H-Bonding configurations.*

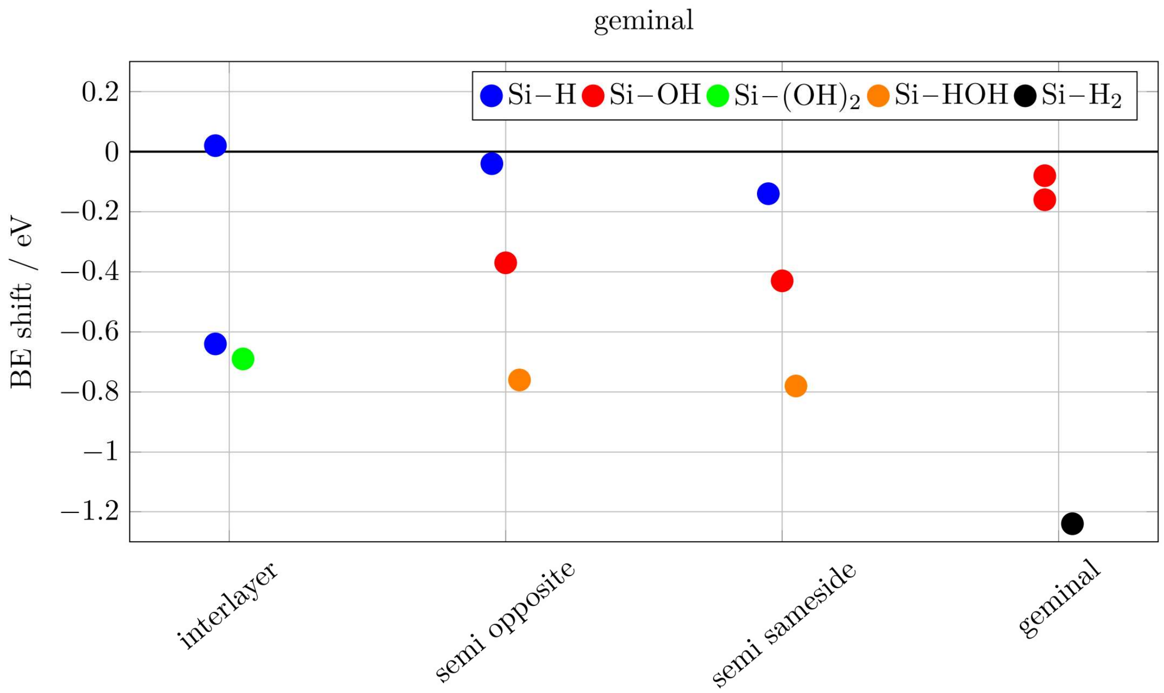

Figure S16. *Bond-saturating double addition of H_2. Geminal Si 2p BE shift values for selected atoms.*

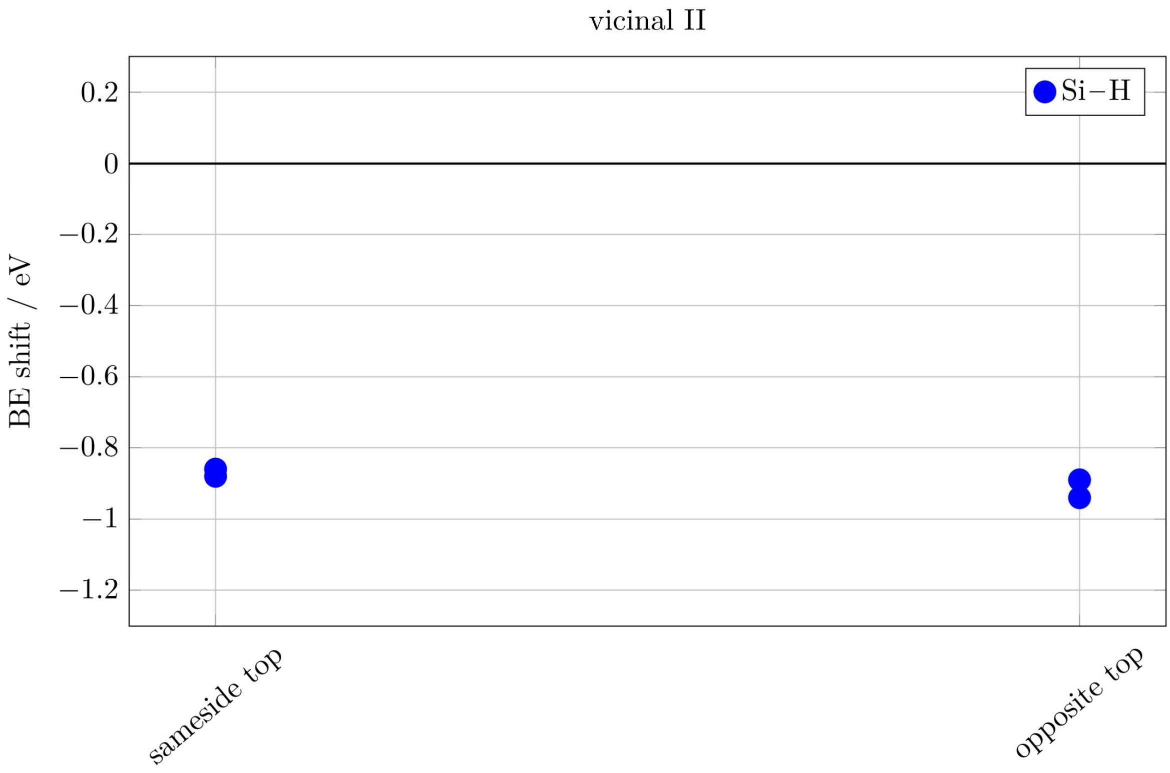

Figure S17.*Vicinal II Si 2p BE shift values for selected atoms.*

3.3 IR Spectra

3.3.1 Pristine bilayer

The IR spectrum of the pristine, crystalline SiO_2 bilayer adsorbed on a (0001) ruthenium surface is shown in Fig. S18. It features the distinctive peak of the bilayer bridging oxygen stretch at 1303cm^{-3}.

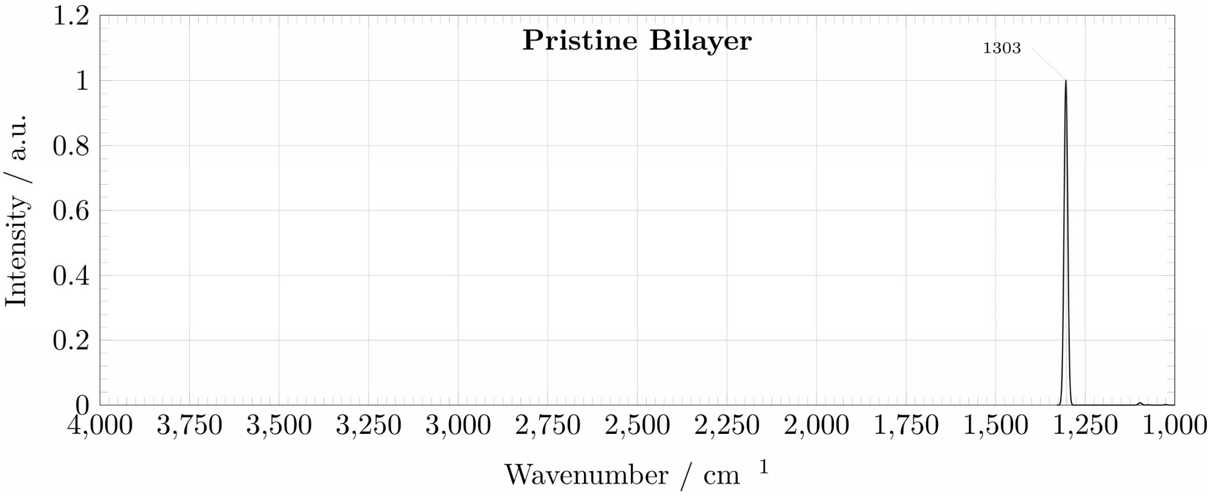

Figure S18. *The IR absorption spectrum for the pristine bilayer, calculated using the z-component of the dipole-moment derivative only.*

5.3.2 Defective bilayer

The following figures show the IR spectra calculated for the structures discussed above, where all hydrogen atoms have been replaced with deuterium atoms. For all calculations, the ruthenium atoms were excluded in the calculation of the Hessian matrix and dipole moments, i.e. they act as a rigid support material. The figures are in order of appearance:

- Bond-saturating single-D_2 - Figure S19
- Vicinal I - Figure S20
- Geminal - Figure S21
- Vicinal II - Figure S22

In general, the plots for the defect structures feature the O–D stretching modes at around 2450 – 2800 cm^{-1}, Si–D stretching modes at around 1550 – 1700 cm^{-1} and a strong peak of the interlayer oxygen stretching mode slightly below 1300 cm^{-1}. Comparing the latter to the frequency of the Si–O–Si vertical stretch of the pristine bilayer, one can see a redshift for this mode by 15 – 30 cm^{-1}, for all defects considered.

For the double D_2 and vicinal II type defects, the deviation in the chemical environment for the different OD or SiD groups results in a splitting of the O–D and/or Si–D bands. The only exceptions are geminal – inside and vicinal II – same side geometries.

In general, a hydroxyl group in the top layer is only able to interact via hydrogen bonding if both OD groups are located on the top layer, while OD groups in the bottom layer may also interact with the interlayer oxygen atoms of the silica bilayer. Consequently, the top O–D

stretching mode is largely independent of other defects, while the bottom mode does show a dependence.

More subtle effects are observed for the structural isomers of the same type, where different locations of the OD groups also influence the position of the Si–D stretching mode. This results, for example, in the "inversion" of the Si–D stretching modes, observed in the vicinal I – crossed case or the splitting of the degenerate Si–D stretching mode in the vicinal I – stacked case.

3.3.3 Bond-saturating single D_2 type defects

The main difference between the bond-saturating single D_2 and the other defects is that there is exactly one Si–D group and one OD group per unit cell (rather than two or none). Consequently, there is exactly one peak for the stretching mode of each group in the spectrum. However, for some of the structures (both-bottom, both-top and both-top-flipped), these peaks are extremely weak, as the defects there are oriented parallel to the surface and therefore a virtually zero dipole change occurs along the *z*-coordinate.

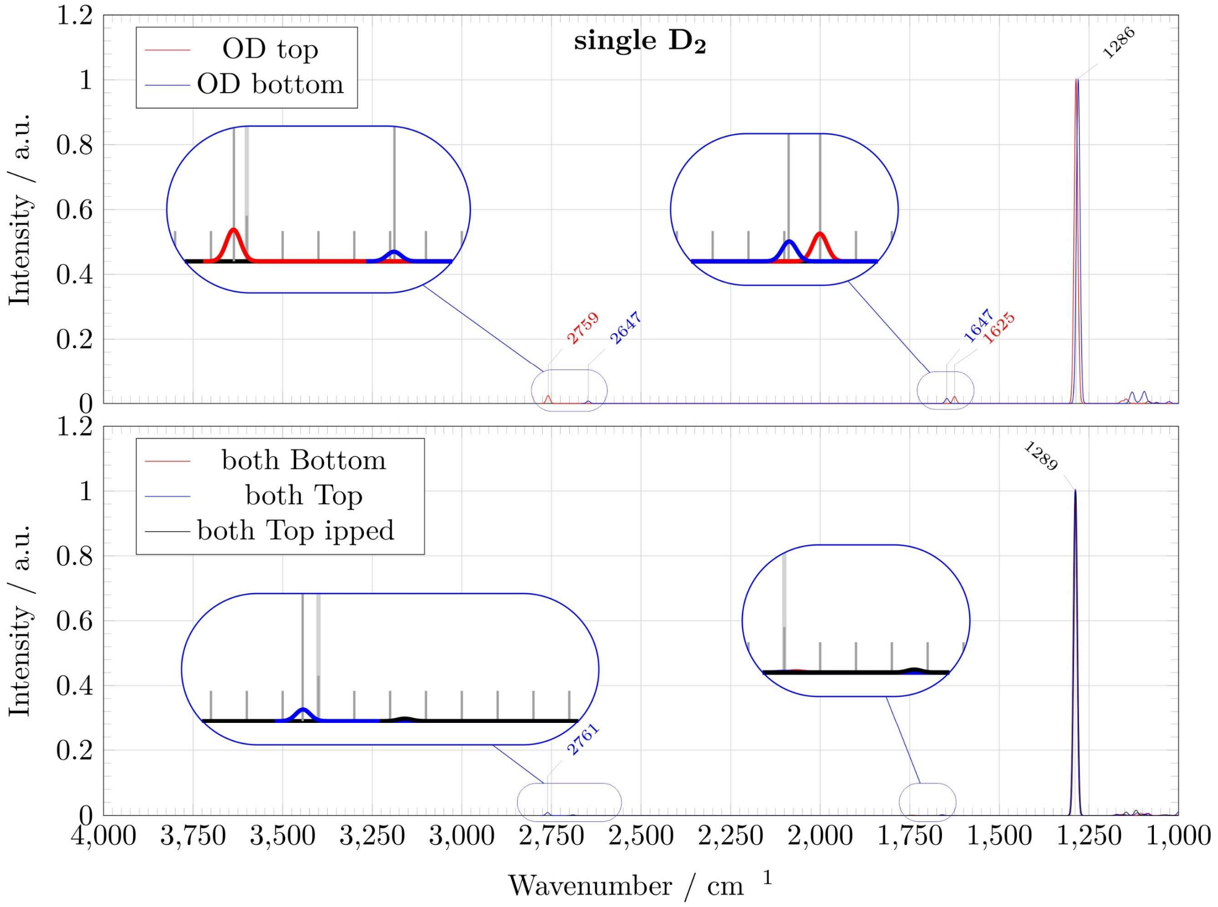

***Figure S19**. The IR absorption spectrum for the different bond-saturating single D_2 geometries under consideration with all hydrogen atoms replaced by deuterium atoms, calculated using the z-component of the dipole-moment derivative only).*

3.3.4 Vicinal I type defects

The vicinal I type defect consists of two OD and two deuterium terminated silicon atoms, resulting from the interaction with two deuterium molecules on the intralayer oxygen bonds. From the six different geometries under consideration, the so-called same side – 1 is different from the crossed and stacked configurations, as it features twice the same defect in one layer, while for the others, the two defect types are always distributed evenly across the top and bottom layer.

This property is also observed in the corresponding infrared spectra (Fig. S20 bottom), where the peaks corresponding to the O–D stretching mode are redshifted considerably between the two isomers same side – 1 and same side – 2, while for the other structures, they are largely comparable.

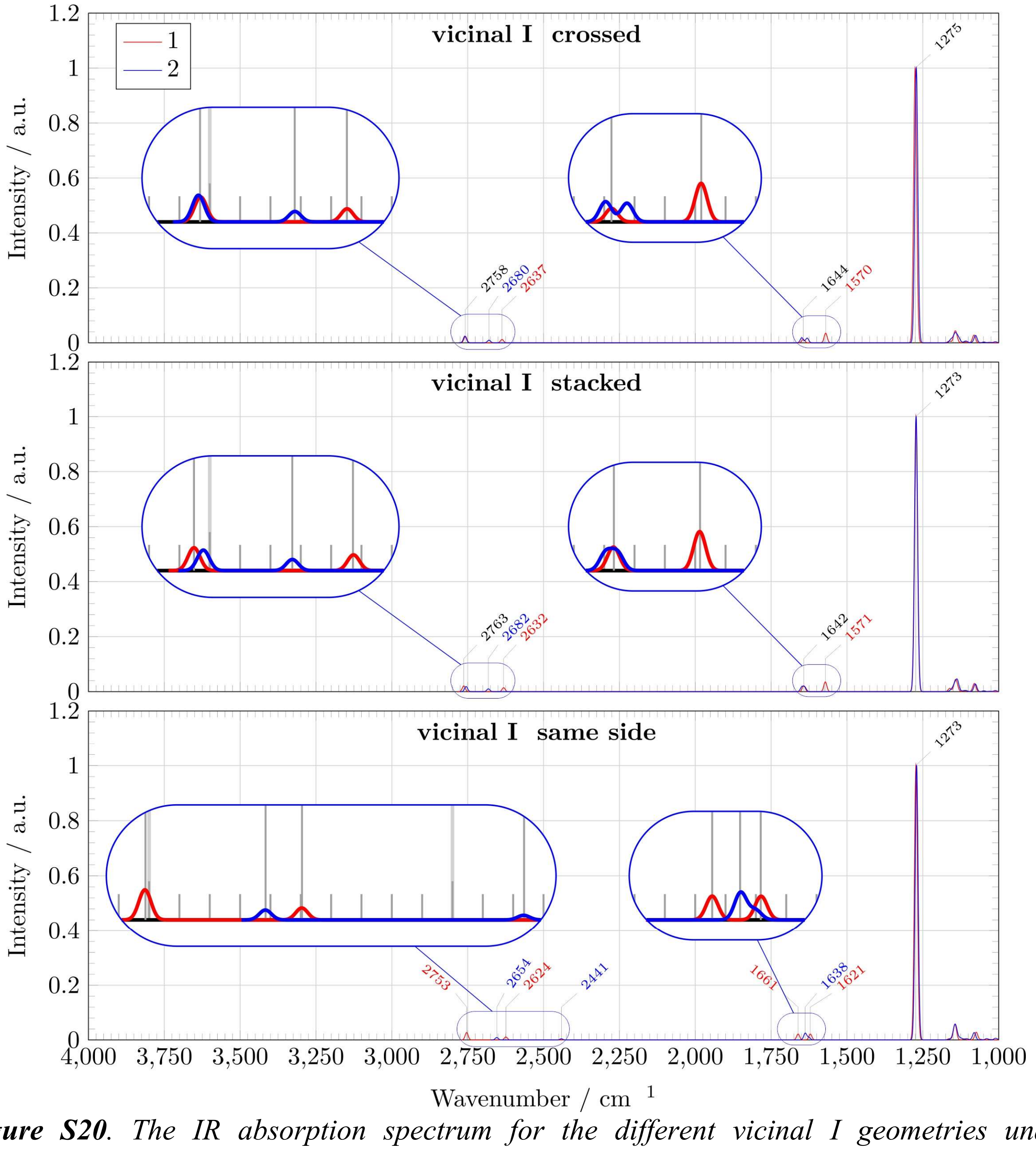

***Figure S20**. The IR absorption spectrum for the different vicinal I geometries under consideration with all hydrogen atoms replaced by deuterium atoms, calculated using the z-component of the dipole-moment derivatives only).*

3.3.5 Geminal type defects

Similar to the vicinal I type defects, the geminal structures are formed by hydrogenation of two Si–O–Si-intralayer bonds, however in this instance, one of the participating silicon atoms carries both defects. Due to the alignment of the OD-groups on the upper defect with the *xy*-plane, there is almost no change in the *z*-component of the dipole moment vector when stretching this bond. Consequently, the intensities in the IR spectrum are extremely low.

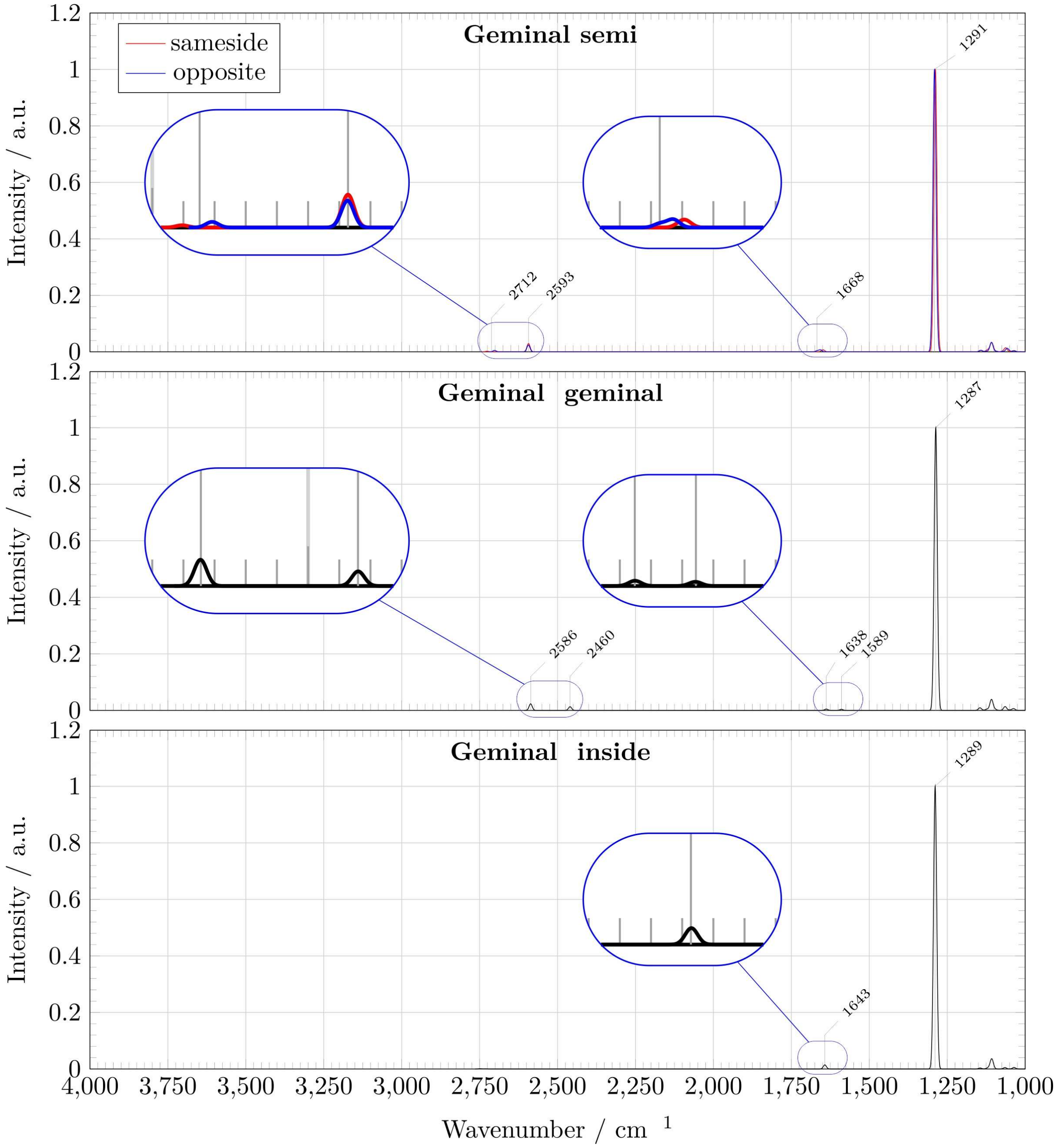

Figure S21. *The IR absorption spectrum for the different geminal type defects under consideration with all hydrogen atoms replaced by deuterium atoms, calculated using the z-component of the dipole-moment derivatives only).*

3.3.6 Vicinal II type defects

For the vicinal II type geometries, the additional bilayer-substrate bonds alter the chemical environment considerably. Additionally, due to the absence of hydroxyl groups in these structures, the corresponding bands are entirely missing in the spectrum (Fig. S22).

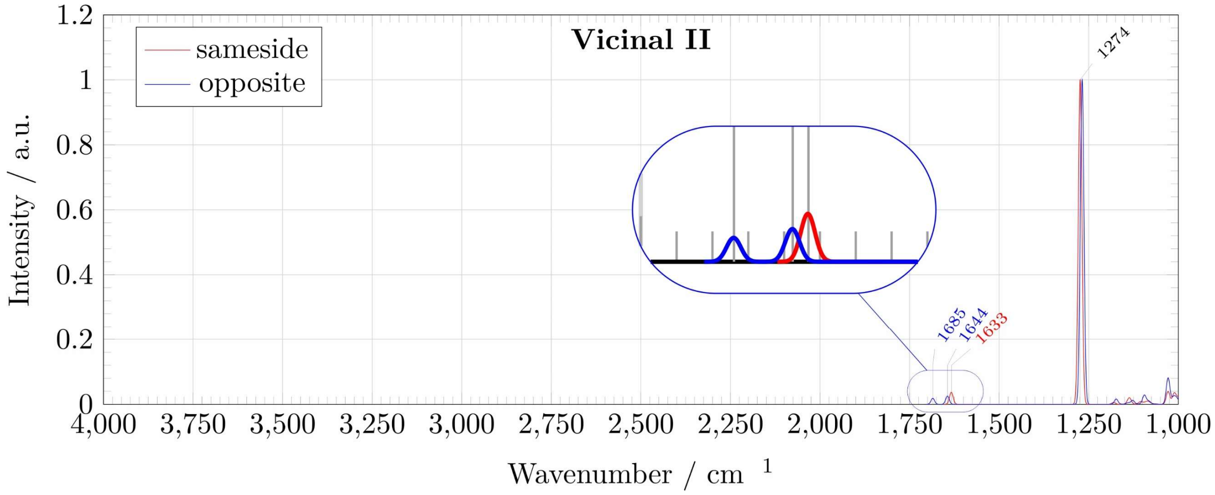

Figure S22*. The IR absorption spectrum for the different Vicinal II geometries under consideration with all hydrogen atoms replaced by deuterium atoms, calculated using the z-component of the dipole-moment derivatives only).*

3.4. Relative energies

In order to analyse the defects from an energetic point of view, we compare DFT total energies for all optimized defect structures considered. Table S1 summarizes the relative electronic stability for both types of defects considered. For the double H_2 defects, the geminal type structures are energetically the most preferable. All of them lie within a 0.1 eV (2.3 kcal/mol) range with respect to the global minimum. The vicinal I structures are somewhat higher, which does not render them inaccessible, as kinetic hinderance may still prevent these structures from finding lower configurations. Nonetheless, the energy difference for most of the vicinal II type structures is above the geminal type by about 0.3 eV (7 kcal/mol) or more.
As for the single H_2 defects, the energetically lowest structures are “OH top” and “both top – flipped”. The “OH bottom” and “both bottom” structures are somewhat higher in energy. The “both-top” and especially the vicinal II type structures are substantially higher, which suggests that their presence is most unlikely.

Table S1. *Relative electronic energies for the structures under consideration.*

	Structure	E_{rel} / eV
**Single H_2 defects**		
Single D_2	OH top	0.01
	OH bottom	0.26
	Both top	0.80
	Both top flipped	0.00
	Both bottom	0.32
Vicinal II	Same side	1.14
	opposite	1.20
**Double H_2 defects**		
Geminal	Geminal	0.08
	Inside	0.09
	Opposite	0.07
	Same side	0.00
Vicinal I	Crossed 1	0.29
	Crossed 2	0.38
	Same side1	0.20
	Same side 2	0.13
	Stacked 1	0.31
	Stacked 2	0.36

4. Kinetic analysis of the hydroxylation process

Based on the simple model that one formed pair of Si-OH and Si-H blocks the three involved pores for further hydroxylation one can describe the surface as an arrangement of dense packed unit cells as shown in Fig. S23.

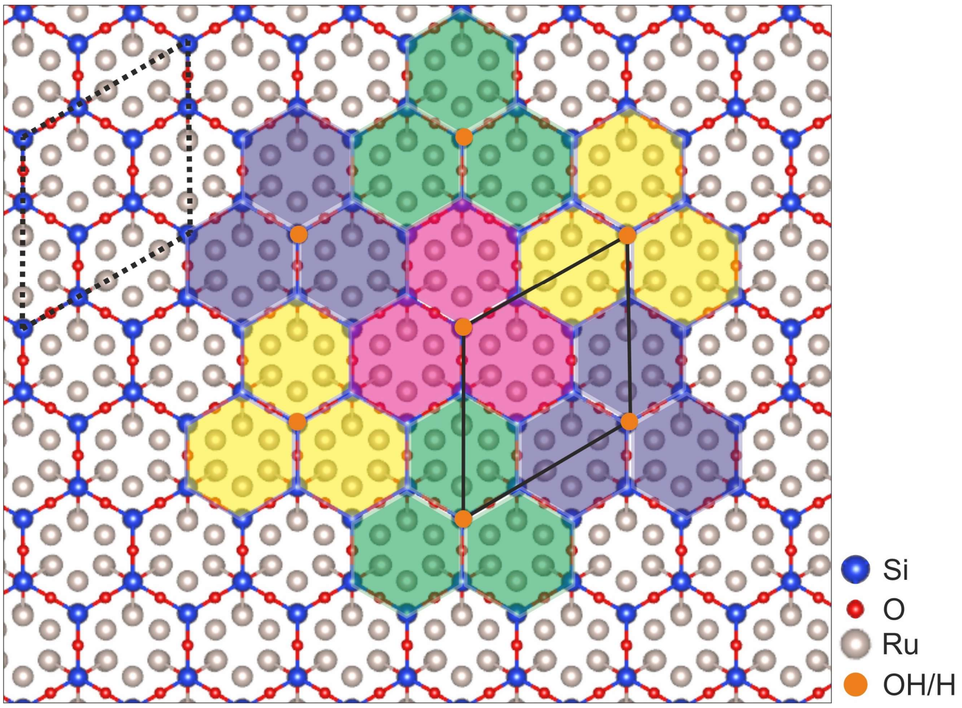

Figure S23. *Ball model describing the possible sites for functionalization of the crystalline SiO2 bilayer supported on Ru(0001). Color-coded hexagons represent the pores of the silica film. Solid and dashed lines indicate the* $(2\sqrt{3} \times 2\sqrt{3})$ *superlattice of the OH/H functional groups, with respect to the Ru(0001) lattice.*

The size of this blocking unit cells is six times the unit cell size of the underlying Ru(0001) surface $A_{blocked} = 12\ A_{Ru}$ giving a saturation density of is $n_{sat} = {}^{1}/_{A_{blocked}} = 1.32\ \mathrm{nm}^{-2}$. Therefore, the hydroxylation process follows a rate equation:

$$\frac{dn}{dt} = -\varrho R(n_{sat} - n) \quad \text{(eq. S1)}$$

meaning the temporal change $\frac{dn}{dt}$ of the hydroxyl concentration n equals a fraction ϱ of the landing rate R, defined as rate of plasma activate H_2 landing on the unit cell $A_{blocked}$, multiplied with the concentration of free unblocked sited $(n_{sat} - n)$. The well-known solution is:

$$n = n_{sat}\left(1 - e^{-t/\tau}\right) \quad \text{(eq. S2)}$$

Fitting the experimental curve in Fig. 2d yields the time constant $\tau = {}^{1}/_{\varrho R} = 47\ s$. The initial hydroxylation rate is:

$$R_0 = \frac{dn}{dt}(t = 0) = \frac{n_{sat}}{\tau} = \ 0.028\ \mathrm{nm}^{-2}\ \mathrm{s}^{-1} \quad \text{(eq. S3)}$$

Furthermore, the landing rate can be estimated by the measured H_2^+ current of $I = 0.5$ µA = -3.125 × $10^{12}\ {}^{e^-}/_{s}$ (with e being the elementary electric charge). With a plasma beam size of d = 7 mm diameter, giving an area of $A_0 = 38.5\ \mathrm{mm}^2$, the H_2^+ flux density can be roughly estimated as:

$$j_{H_2} = \frac{I/_{e^-}}{A_0} = 8.1 \times 10^{10}\ \mathrm{mm}^{-2}\ \mathrm{s}^{-1} = 0.081\ \mathrm{nm}^{-2}\ \mathrm{s}^{-1} \quad \text{(eq. S4)}$$

With the landing rate being $R = \frac{j_{H_2}}{n_{sat}}$.

Comparing eq. S3 with eq. S4, one finds the fraction ϱ of the flux of incoming H_2^+ used for the hydroxylation:

$$\varrho = \frac{R_0}{j_{H_2}} \approx 0.35 = 35\% \quad \text{(eq. S5)}$$

That means during the initial process the incoming plasma activate H_2^+ are nearly completely used for the hydroxylation (and hydrogenation) of the silica, exhibiting a high reactivity of the species. The rest contributes either to the removal of the interfacial oxygen at the Ru(0001) surface or does not react with the surface.

5. References

1. Bancroft, G. M.; Nesbitt, H. W.; Ho, R.; Shaw, D. M.; Tse, J. S.; Biesinger, M. C., Toward a comprehensive understanding of solid-state core-level XPS linewidths: Experimental and theoretical studies on the Si 2p and O 1s linewidths in silicates. *Phys Rev B* **2009,** *80* (7), 075405.
2. Prieto, M. J.; Mullan, T.; Schlutow, M.; Gottlob, D. M.; Tănase, L. C.; Menzel, D.; Sauer, J.; Usvyat, D.; Schmidt, T.; Freund, H.-J., Insights into Reaction Kinetics in Confined Space: Real Time Observation of Water Formation under a Silica Cover. *J Am Chem Soc* **2021,** *143* (23), 8780-8790.
3. Yu, X.; Emmez, E.; Pan, Q.; Yang, B.; Pomp, S.; Kaden, W. E.; Sterrer, M.; Shaikhutdinov, S.; Freund, H.-J.; Goikoetxea, I.; Wlodarczyk, R.; Sauer, J., Electron stimulated hydroxylation of a metal supported silicate film. *Phys Chem Chem Phys* **2016,** *18* (5), 3755-3764.
4. Köhler, L.; Kresse, G., Density functional study of CO on Rh(111). *Phys Rev B* **2004,** *70* (16), 165405.
5. Włodarczyk, R.; Sierka, M.; Sauer, J.; Löffler, D.; Uhlrich, J. J.; Yu, X.; Yang, B.; Groot, I. M. N.; Shaikhutdinov, S.; Freund, H. J., Tuning the electronic structure of ultrathin crystalline silica films on Ru(0001). *Phys Rev B* **2012,** *85* (8), 085403-1.
